# A Combined *In Vivo* HSC Transduction/Selection Approach Results in Efficient and Stable Gene Expression in Peripheral Blood Cells in Mice

**DOI:** 10.1016/j.omtm.2017.11.004

**Published:** 2017-11-10

**Authors:** Hongjie Wang, Maximilian Richter, Nikoletta Psatha, Chang Li, Jiho Kim, Jing Liu, Anja Ehrhardt, Susan K. Nilsson, Benjamin Cao, Donna Palmer, Philip Ng, Zsuzsanna Izsvák, Kevin G. Haworth, Hans-Peter Kiem, Thalia Papayannopoulou, André Lieber

**Affiliations:** 1 University of Washington, Department of Medicine, Division of Medical Genetics, Box 357720, Seattle, WA 98195, USA; 2 Department of Medicine, Division of Hematology, University of Washington, Seattle, WA, USA; 3 Department of Pathology, University of Washington, Seattle, WA, USA; 4 Witten/Herdecke University, Witten, 58448, Germany; 5 Biomedical Manufacturing, CSIRO, Clayton, VIC 3800, Australia; 6 Australian Regenerative Medicine Institute, Monash University, Clayton, VIC 3800, Australia; 7 Baylor College of Medicine, Houston, TX 77046, USA; 8 Max-Delbrück-Center for Molecular Medicine, Berlin 13092, Germany; 9 Fred Hutchinson Cancer Research Center, Seattle, WA 98109, USA

**Keywords:** hematopoietic stem cells, adenovirus, mobilization

## Abstract

We recently reported on an *in vivo* hematopoietic stem cell (HSC) gene therapy approach. It involves the subcutaneous injections of G-CSF/AMD3100 to mobilize HSCs from the bone marrow into the peripheral blood stream and the intravenous injection of an integrating helper-dependent adenovirus vector system. HSCs transduced in the periphery homed back to the bone marrow, where they persisted long-term. However, high transgene marking rates found in primitive bone marrow HSCs were not reflected in peripheral blood cells. Here, we tested small-molecule drugs to achieve selective mobilization and transduction of HSCs. We found more efficient GFP marking in bone marrow HSCs but no increased marking in the peripheral blood cells. We then used an *in vivo* HSC chemo-selection based on a mutant of the O^6^-methylguanine-DNA methyltransferase (mgmt^P140K^) gene that confers resistance to O^6^-BG/BCNU and should give stably transduced HSCs a proliferation stimulus and allow for the selective survival and expansion of progeny cells. Short-term exposure of G-CSF/AMD3100-mobilized, *in vivo*-transduced mice to relatively low selection drug doses resulted in stable GFP expression in up to 80% of peripheral blood cells. Overall, the further improvement of our *in vivo* HSC transduction approach creates the basis for a simpler HSC gene therapy.

## Introduction

**Figure 1:**
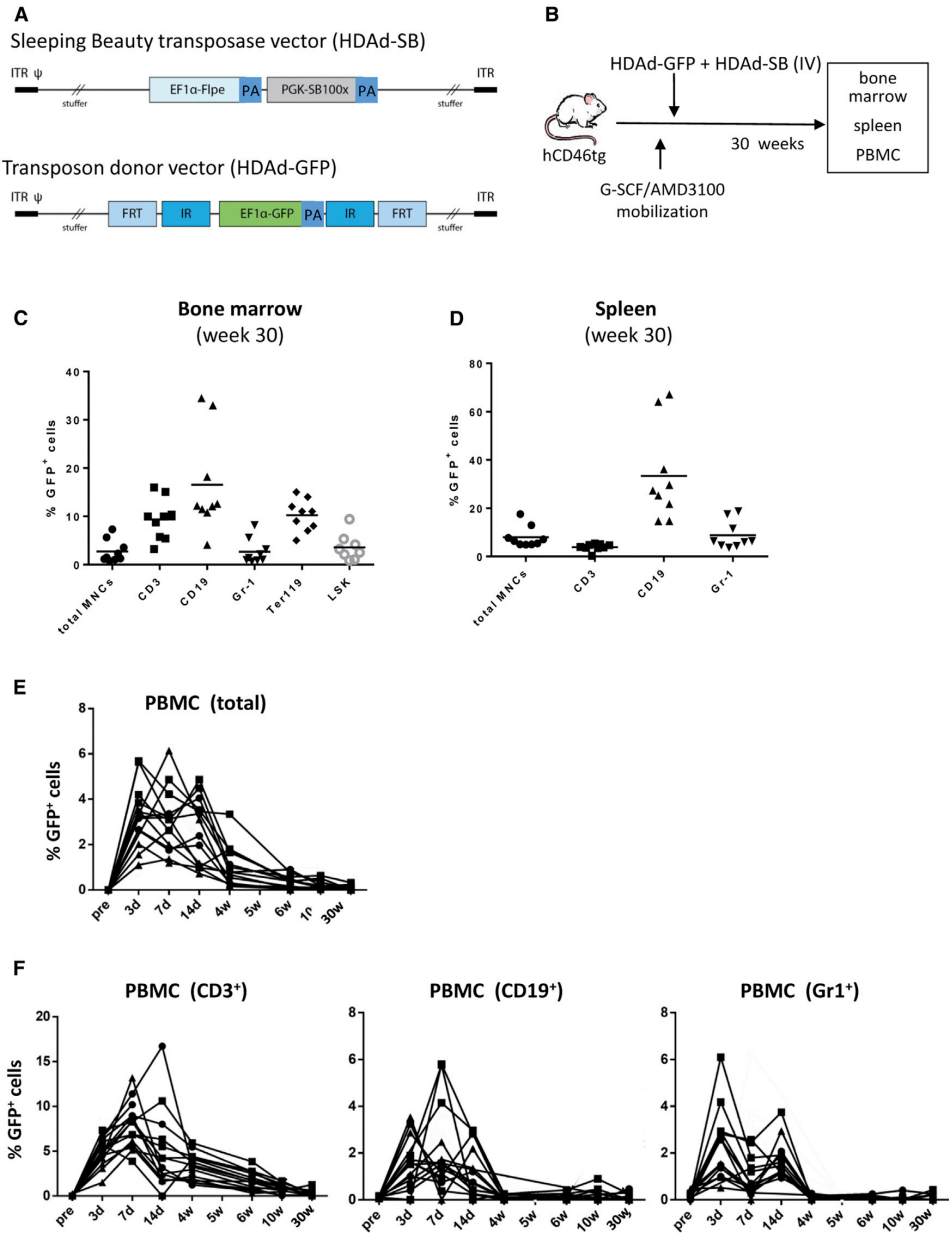
GFP Expression in HSCs and Lineage-Positive Cells in Bone Marrow, Spleen, and PBMCs (A) Integrating HDAd5/35++ vectors. The transposon vector (HDAd-GFP) carries the GFP expression cassette that is flanked by inverted transposon repeats (IR) and FRT sites. PA, polyadenylation signal. The second vector (HDAd-SB) provides both Flpe recombinase and the SB100x transposase in *trans*. Both vectors are helper-dependent (HDAd) adenovirus vectors containing the CD46 affinity-enhanced Ad35++ fiber knob and the Ad35 fiber shaft.^28^ Upon co-infection of both vectors, Flpe mediates the circularization of the transposon through FRT sites. SB100x then randomly integrates the transposon into the host genome through interaction with the IRs. (B) *In vivo* transduction of mobilized hCD46tg mice. HSCs were mobilized by s.c. injection of human recombinant G-CSF for 4 days followed by an s.c. injection of AMD3100. 30 and 60 min after AMD3100 injection, animals were intravenously injected with a 1:1 mixture of HDAd-GFP + HDAd-SB (2 injections, each 4 × 10^10^ vp). Mice were sacrificed at week 30 after HDAd-GFP + HDAd-SB injection. (C) Bone marrow at week 30 after HDAd-GFP injection. Shown is the percentage of GFP^+^ cells in total mononuclear cells (MNCs), lineage-positive cells (CD3^+^, CD19^+^, Gr-1^+^, and Ter119^+^), and HSCs (LSK cells). Each symbol is an individual animal. (D) Spleen. Percentage of GFP^+^ cells in MNCs and lineage-positive cells at week 30. (E) Percentage of GFP^+^ cells in total PBMCs measured at the indicated time points after *in vivo* HDAd injection. Each line is an individual animal. N = 10. (F) Percentage of GFP^+^ cells in peripheral blood lineages.

Current hematopoietic stem cell (HSC) gene therapy protocols are based on the transplantation of *ex-vivo*-transduced HSCs into myelo-conditioned recipients. *Ex vivo* culturing of HSCs limits the ability to transduce the most primitive stem cells, a limitation that can result in the loss of transduced cells over time in transplant recipients. Furthermore, the process of *ex vivo* HSC manipulation/transplantation is expensive and must be performed in specialized, accredited centers, a requirement that severely limits access to patients with common genetic diseases. To simplify HSC gene therapy, we recently developed an approach for *in vivo* HSC transduction. It involves subcutaneous injections of granulocyte colony-stimulating factor (G-CSF)/AMD3100 to mobilize HSCs from the bone marrow into the peripheral blood stream and the intravenous injection of an integrating helper-dependent adenovirus (HDAd5/35++) vector system.^1^ These vectors target CD46, a receptor that is expressed at higher levels in HSCs than in more differentiated bone marrow and blood cells. We demonstrated in transgenic mice expressing human CD46 (hCD46) in a pattern similar to humans^2^ and in immunodeficient mice with engrafted human CD34^+^ cells that HSCs transduced with HDAd5/35++ in the periphery home back to the bone marrow, where they persist and stably express the transgene long-term.^1^ To confer integration of a GFP transgene cassette, we utilized a hyperactive Sleeping Beauty transposase (SB100x) system^3^ in the context of a helper-dependent HDAd5/35++ vector (HDAd-SB) (Figure 1A). In our previous study,^1^ at 20 weeks after mobilization and intravenous injection of a EF1α-promoter-GFP-cassette-containing transposon vector (HDAd-GFP) and HDAd-SB, we detected GFP marking in bone marrow lineage(lin)^−^/Sca1^+^/cKit^+^ (LSK) cells in the range of 5% and in colony-forming units (CFUs) in the range of 20%. However, the percentage of GFP-expressing peripheral blood mononuclear cells (PBMCs) was on average less than 1% at 20 weeks post-transduction. This is a shortcoming of our approach because for most genetic blood disorders to be cured, the transgene product must be expressed in differentiated peripheral blood cells.

To improve upon this shortcoming, we pursued two different strategies aimed at increasing the frequency of transgene-expressing peripheral blood cells. The first approach is based on the assumption that G-CSF/AMD3100 mobilization with subsequent HDAd5/35++ *in vivo* transduction does not allow for the transduction of a sufficiently high number of HSCs. So far, we have used G-CSF and AMD3100 for HSC mobilization because this approach is broadly used for HSC collection.^4^ G-CSF stimulates proliferation of cells in bone marrow and spleen and results in mobilization of not only HSCs but also less primitive progenitors into the peripheral blood circulation, leading to a general increase in white blood cells, i.e., targets for HDAd5/35++ transduction. This “sponge” effect reduces the effective vector dose capable of transducing HSCs. Therefore, we evaluated alternative HSC mobilization agents in hCD46 transgenic mice. HSC mobilization can be achieved by interfering with either (1) α_4_β_1_ (VLA) and α_9_β_1_ integrins binding to vascular cell adhesion molecule 1 (VCAM1) or (2) interactions between the chemokine receptor CXCR4 and its ligand SDF-1. AMD3100, a synthetic small-molecule CXCR4 antagonist, leads to more rapid mobilization of HSCs than does G-CSF^5^, ^6^, ^7^ and causes mobilization solely through disruption of the SDF-1-CXCR4 axis. Other small-molecule drugs that act by mechanisms different from AMD3100 are BIO5192 and BOP (N-(benzenesulfonyl)-L-prolyl-L-O-(1-pyrrolidinylcarbonyl)tyrosine. BIO5192 is a small-molecule VLA-4 inhibitor.^8^ BOP is a small molecule targeting α_9_β_1_/α_4_β_1_ integrins.^9^

The second approach is based on our previous conclusion that *in vivo*-transduced HSCs are, per se, capable of differentiating. For example, bone marrow GFP^+^ mononuclear cells harvested at 16 weeks after *in vivo* transduction repopulated all peripheral blood cell lineages after transplantation into lethally irradiated recipients.^1^ To stimulate cell division of GFP^+^ HSCs and give their progeny a selective advantage, we plan to use a drug-resistance gene followed by application of the appropriate cytotoxic agent.^10^ Upon transplantation of lentivirus vector transduced HSCs, *in vivo* chemo-selection has been successfully demonstrated using a P140K mutant version of the human O^6^-methylguanine-DNA methyltransferase (mgmt) gene that confers resistance to relatively low-doses of methylating agents (e.g., O^6^-benzylguanine [O^6^-BG] plus bis-chloroethylnitrosourea (BCNU) or temozolomide.^11^, ^12^, ^13^ MGMT^P140K^ expression in HSCs allowed for stable multi-lineage *in vivo* selection in mice, dogs, non-human primates, and humans.^11^, ^14^, ^15^ Here, we incorporated a mgmt^P140K^ gene into the HDAd-GFP vector and assessed GFP marking in *in vivo*-transduced hCD46 transgenic mice.

## Results

### Discrepancy in Transgene Marking in Bone Marrow HSCs and Differentiated Peripheral Blood Cells

We transduced G-CSF/AMD3100 mobilized hCD46tg mice with HDAd-GFP + HDAd-SB (Figures 1A and 1B). We periodically measured GFP expression in PBMCs and sacrificed the animals 30 weeks later to analyze GFP expression in bone marrow and spleen. In bone marrow, GFP marking in LSK cells, a subfraction that is considered to be enriched for HSCs, was ∼5% (Figure 1C). Average GFP marking rates in the range of 10%–18% were found in bone marrow CD3^+^ and CD19^+^ cells as well as in Ter119^+^ cells. The percentage of GFP-expressing pro-granulocytes (Gr-1^+^) in bone marrow was ∼3%. In the spleen, a secondary hematopoietic organ to which mobilized HSCs can return,^1^ we found GFP marking in ∼10% of CD3^+^ and Gr-1^+^ cells (Figure 1D). Around 30% of CD19^+^ cells in the spleen expressed GFP. In contrast to marking rates in bone marrow, GFP expression in PBMCs at 30 weeks was less than 1% in all animals (Figure 1E). Looking at the kinetics of the percentage of GFP^+^ PBMCs, the first 2 weeks show GFP marking in 2%–6% of PBMCs which is in agreement with our previous study,^1^ and most likely originates from direct transduction of circulating PBMCs in the peripheral blood after intravenous injection of HDAd-GFP + HDAd-SB. The slow decline of GFP^+^ PBMCs by week 10 could be the results of natural turnover of transduced cells. Within transduced PBMCs, CD3^+^ T cells represented the largest fraction (up to 15% at week 2) (Figure 1F).

#### Approach 1: More Efficient *In Vivo* Transduction of HSCs through Selective HSC Mobilization Agents

**Figure 2:**
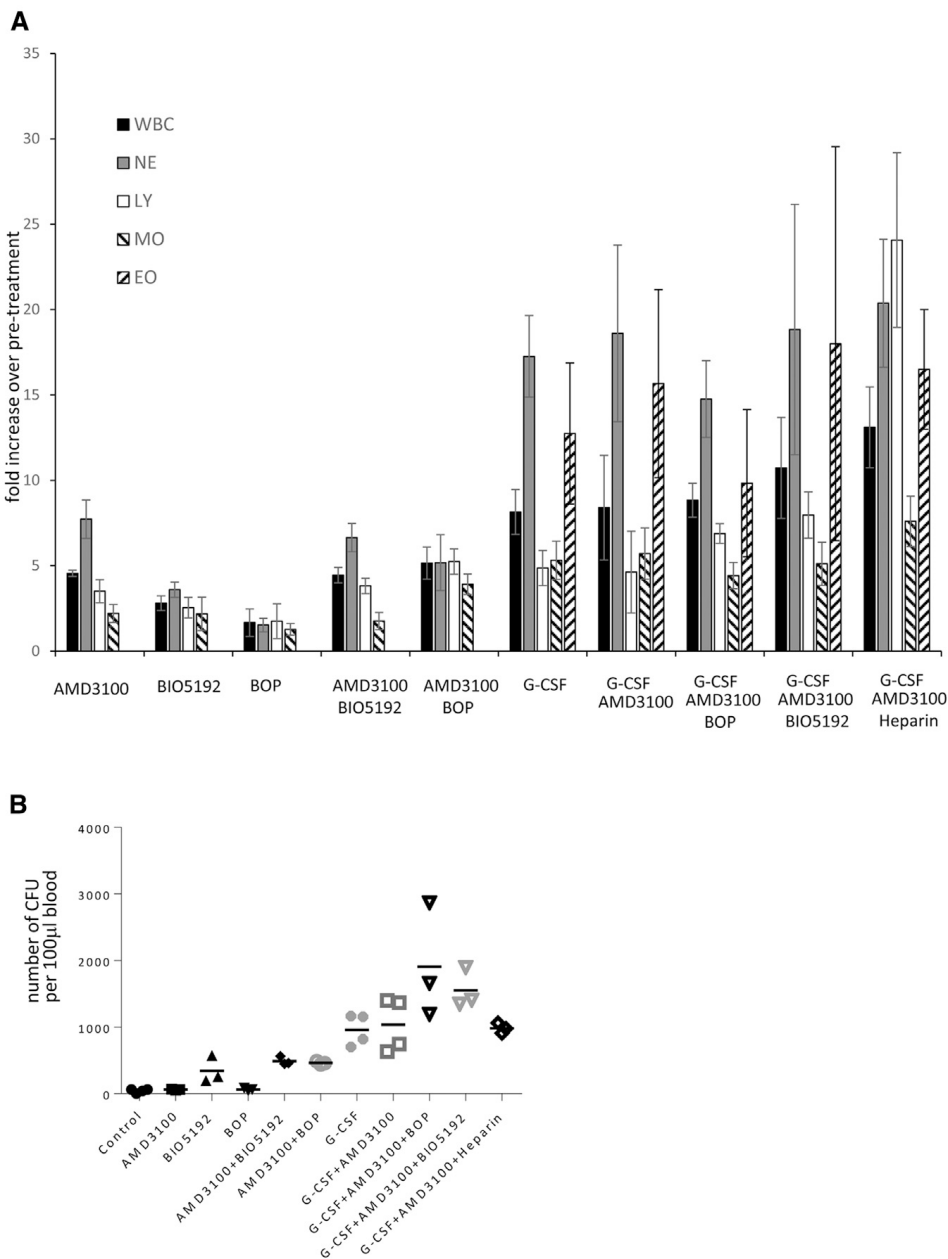
Effect of Different Mobilization Drugs on White Blood Cell Counts and Colony-Forming Cells in Peripheral Blood 1 hr after the Last Drug Injection (A) Peripheral white blood cell counts after mobilization. G-CSF was given s.c. daily for 4 days. For combinations with G-CSF, drugs were given at day 5. The following doses were administered: G-CSF (5 μg/mouse, s.c.), AMD3100 (5 mg/kg, s.c.), BIO5192 (1 mg/kg, i.v.), BOP (10 mg/kg, s.c.), and heparin (100 units, i.p., together with AMD3100). Peripheral blood samples were collected 1 hr after the last drug injection. Shown is the fold increase in white blood cells over pre-treatment levels. N = 3 animals. WBC, white blood cells; NE, neutrophils; LY, lymphocytes; MO, monocytes; EO, eosinophils. (B) Mobilization of HSCs based on the number of progenitor colony-forming cells (CFCs) in 100 μL of blood. Blood samples were treated with red cell lysis buffer, and all leukocytes were plated. Colonies were counted 12 days later. Each symbol is one animal. “Control” refers to untreated animals.

We compared the mobilization efficiency of different agents based on white blood cell numbers and HSCs in peripheral blood at 1 hr after drug injection. The goal of this study was to find an approach that would result in more selective and/or efficient HSC mobilization. As expected, G-CSF alone and all combinations that contained G-CSF resulted in high leukocytosis with particularly strong increases (>15-fold) in neutrophil and eosinophil numbers (Figure 2A). AMD3100, BIO5192, and BOP alone or in combination increased white blood cells counts only between 2- and 5-fold, with BOP triggering the least leukocytosis.

Hematopoietic progenitor numbers in the peripheral blood were first measured by a functional HSC assay, i.e., the ability to form multi-lineage progenitor colonies. Peripheral blood was collected 1 hr after the last drug injection and white blood cells were plated for CFU assays (Figure 2B). Overall, G-CSF (alone and in combination) mobilized CFU more efficiently than settings without G-CSF (p < 0.05 compared to control). There was a trend that the addition of BOP or BIO5192 to G-CSF/AMD3100 increased the mobilization efficacy but the difference was not significant (p = 0.061 and p = 0.07). The addition of heparin to G-CSF/AMD3100 did not increase the number of mobilized CFU and increased spontaneous bleeding. AMD3100 and BOP alone had only marginal effects on peripheral blood CFU numbers. However, BIO5192 and the combinations of AMD3100/BIO5192 and AMD3100/BOP resulted in some degree of HSC mobilization (p < 0.02 for all three). The mobilization effect of AMD3100 alone was also analyzed by flow cytometry for the LSK cell phenotype of HSCs (Figure S1). A single injection of AMD3100 did not increase the percentage of LSK cells in the peripheral blood at 1 and 3 hr after injection, while G-CSF alone did. We did not test a recently published approach that involved a continuous infusion of AMD3100 over 2 weeks and resulted in efficient HSC mobilization.^7^

Because of leukocytosis associated with G-CSF, further studies have focused on AMD3100, BIO5192, and BOP alone compared to the standard regimen.

##### GFP Marking at 8 Weeks after HDAd-GFP + HDAd-SB IV Injection of Mice that Were Mobilized with AMD3100, BIO5192, and/or BOP

**Figure 3:**
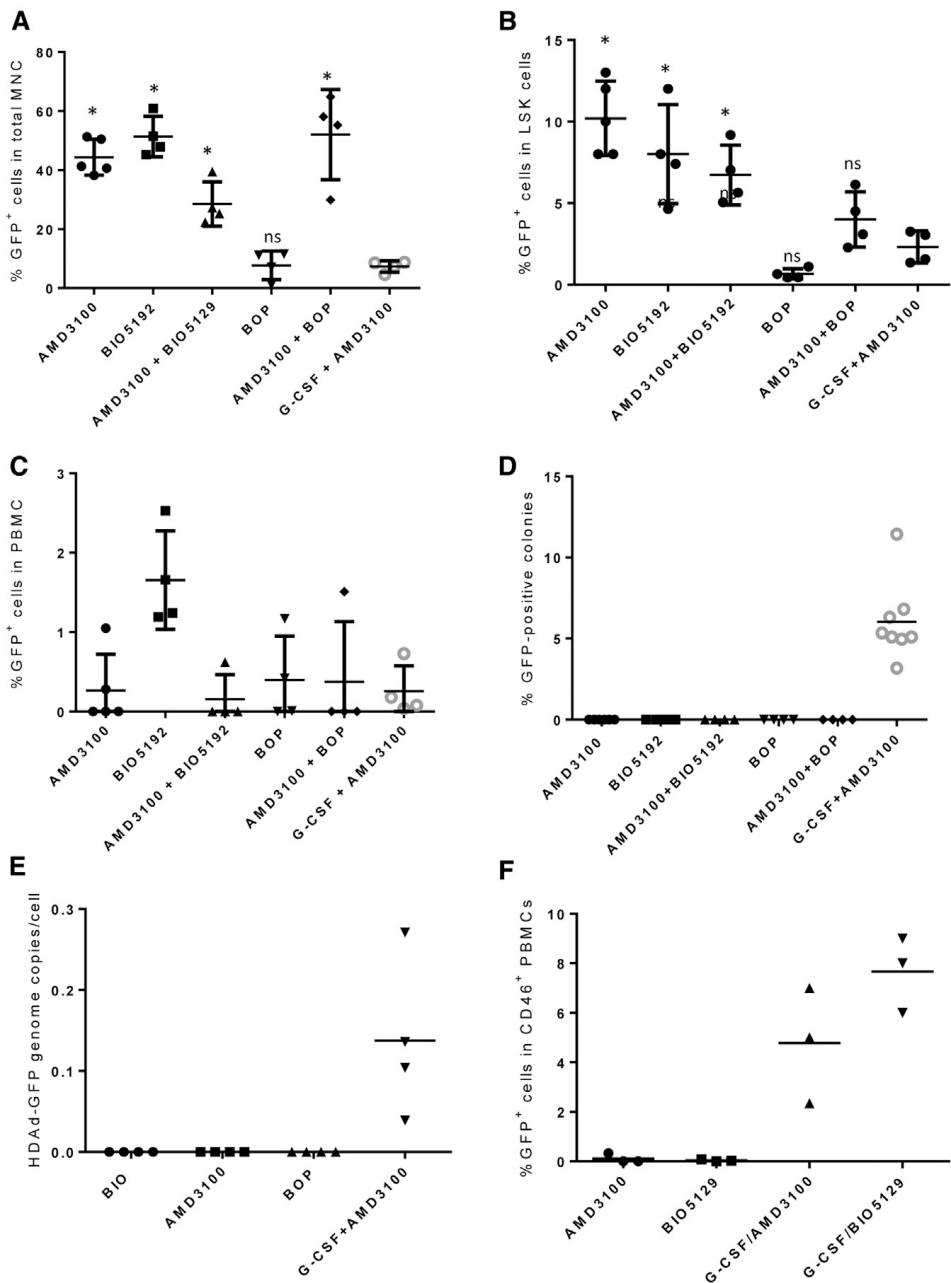
GFP Analysis at 8 Weeks after *In Vivo* Transduction with HDAd-GFP and HDAd-SB and Different Mobilization Regimens (A–F) hCD46tg mice were mobilized with the indicated agents and i.v. injected with HDAd-GFP + HDAd-SB. (Notably, the vector dose [2 times, 2 × 10^10^ vp] was half of that used in our previous studies^1^ and in the studies shown in Figure 1.) Mice were sacrificed at 8 weeks after injection, and bone marrow cells, splenocytes, and PBMCs were harvested. (A) GFP expression in total bone marrow mononuclear cells (MNCs). (B) Percentage of GFP^+^ cells in LSK cells. (C) Percentage of GFP^+^ PBMCs. (D) Colony assay. Bone marrow MNCs were depleted for lineage-positive cells, sorted for GFP^+^ cells, and plated for progenitor colonies assays. Shown is the percentage of GFP^+^ colonies that formed after 14 days. (E) HDAd-GFP vector DNA copies in pooled colonies. qPCR with primers specific to the GFP cassette using genomic DNA from pooled colonies derived from plated lin^−^/GFP^+^ cells. Each symbol is the result of pooled colonies from a given animal. (F) Transplantation assay. Bone marrow cells harvested from *in vivo*-transduced hCD46 transgenic mice were transplanted into C57BL/6 mice. Shown is the percentage of GFP^+^ cells in hCD46^+^ PBMCs at week 8 after transplantation. *p < 0.05.

At week 8 after *in vivo* transduction, the composition of lin^+^ cells in bone marrow, spleen, and blood was similar to non-mobilized animals (Figure S2). In bone marrow, AMD3100 and BIO5192 mobilization alone or in combination resulted in ∼50% GFP^+^ mononuclear cells (MNC) (p < 0.01 compared to G-CSF/AMD3100) (Figure 3A). GFP expression in BOP-only mobilized mice was comparable to the G-CSF/AMD3100 setting. A similar trend was seen for bone marrow LSK (Figure 3B) and lin^+^ cells (Figure S3). In the spleen, AMD3100 or BIO5192 mobilized animals also had higher GFP marking (Figure S4). Interestingly, the combination of AMD3100 and BIO5192 was less efficient suggesting that this drug combination either interfered with the expansion of directly transduced splenic cells, the return of mobilized cells to the spleen, or the influx of modified, BM-derived cells.

Despite the high marking rates obtained with AMD3100 and BIO5192 in bone marrow and spleen, which indicate more efficient/selective transduction of potential HSCs and progenitors in the periphery and/or return to hematopoietic tissues, the percentage of GFP^+^ PBMCs was below 1.5% for all mobilization settings (Figure 3C). Moreover, we found that GFP marking seen at week 8 in AMD3100 and BIO5192 mobilized animals originated from episomal vectors and was lost once proliferation was triggered in transduced cells either in CFU or in transplantation assays (Figures 3D–3F). In colony assays, the frequency of colony formation from plated bone marrow lin^−^/GFP^+^ cells was comparable for all groups (Figure S5). However, although all the plated cells were GFP^+^, GFP^+^ colonies were observed only in G-CSF/AMD3100-mobilized mice (Figure 3D). To test whether this is due to failure of vector integration and subsequent loss of episomal vector DNA during proliferation, we measured vector DNA copy numbers by qPCR with GFP-specific primers in pooled colonies (Figure 3E). The analysis demonstrated the absence of vector DNA in colonies from BIO5192-, AMD3100-, and BOP-treated mice. In contrast, the vector DNA signal correlated with GFP expression in pooled colonies from G-CSF/AMD3100-mobilized mice. Notably the vector copy number measured in lin^−^/GFP^+^ cells before plating was comparable in all settings (∼1.3 copies per cell). The lack of detectable integration in AMD3100- or BIO5192-mobilized, *in vivo*-transduced animals was also evident in transplantation studies, where bone marrow cells from *in vivo*-transduced hCD46tg mice were transplanted into C57BL/6 mice. Engraftment was comparable for all groups (∼95%) (Figure S6). However, only mice mobilized with G-CSF/AMD3100 showed GFP-expressing PBMCs (Figure 3F). This indicates that although ∼40% of bone marrow MNCs expressed GFP in the transplant, GFP expression was lost after engraftment and differentiation of transduced HSCs.

In summary, AMD3100 and BIO5192 mobilization in combination with HDAd vector injection allows for efficient transient GFP marking in bone marrow MNCs, including LSK cells at week 8. This supports our hypothesis that more selective mobilization by these drugs increases the transduction of certain bone marrow subsets. However, our data also indicate that SB100x-mediated transposon integration requires the effects of G-CSF on mobilized cells. With regard to increasing GFP marking in PBMCs, our studies with AMD3100, BIO5192 and BOP failed to achieve this at a level that would be therapeutically meaningful. All subsequent *in vivo* HSC transduction studies were therefore performed with G-CSF/AMD3100 mobilized hCD46tg mice.

#### Approach 2: Trigger Cell Cycling in Quiescent GFP^+^ HSCs and Provide Proliferative Advantage to Transduced HSC Progeny in Bone Marrow

**Figure 4:**
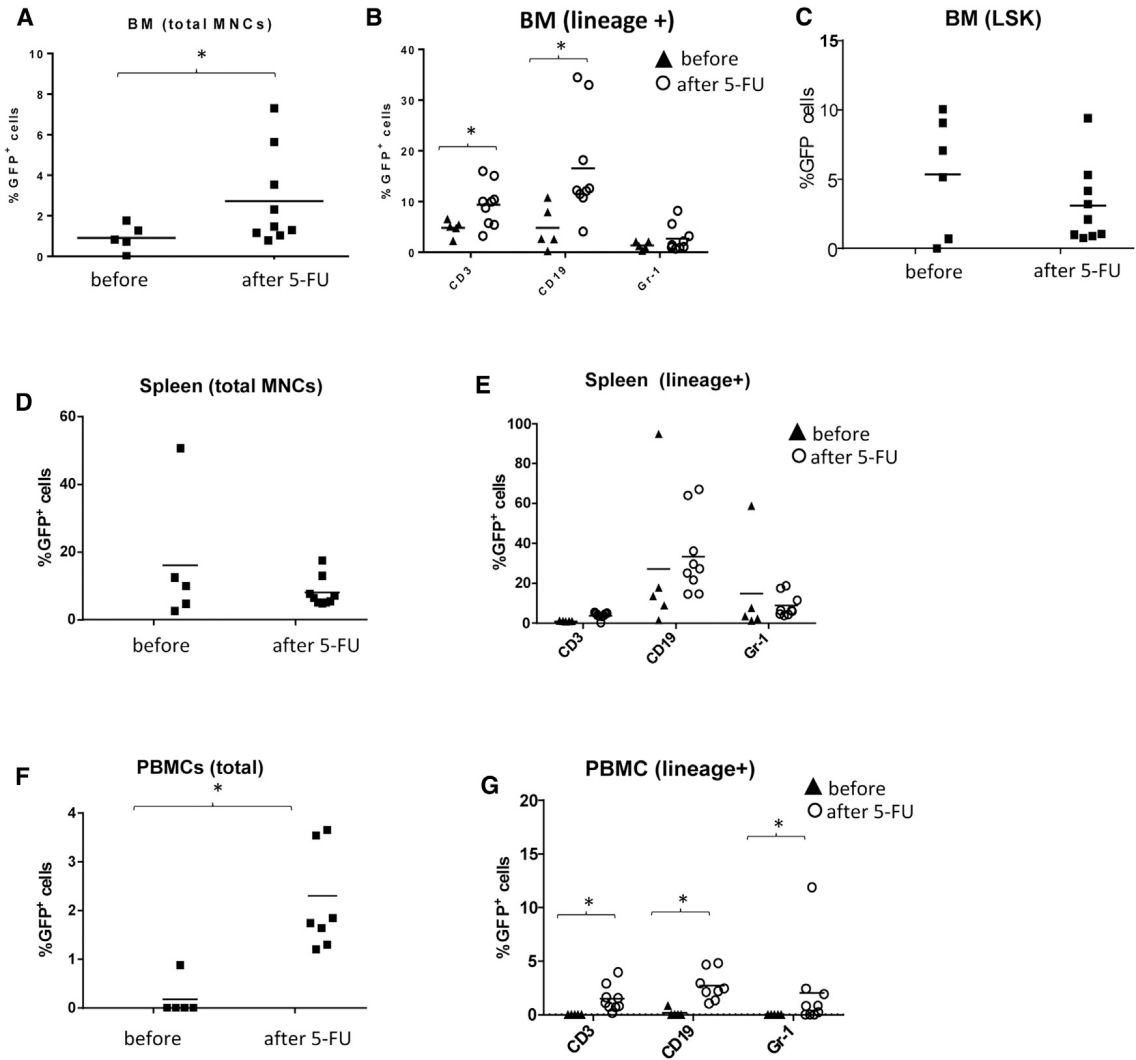
Comparison of GFP Marking in Bone Marrow, Spleen, and PBMCs before the Start of 5-FU Treatment (Week 20 after *In Vivo* Transduction) and after 10 Weeks of Weekly 5-FU Treatment (A–C) G-CSF/AMD3100-mobilized mice were transduced with HDAd-GFP + HDAd-SB as described in Figure 1. Mice were sacrificed at week 30 after HDAd injection after 10 weeks of 5-FU treatment (40 mg/kg, weekly, IP). Bone marrow (BM). Shown is the percentage of GFP^+^ cells in total mononuclear cells (A), T cells, B cells, and myeloid cells (B), and HSC LSK cells (C). (D and E) Spleen. Shown is the percentage of GFP^+^ cells in total spleen MNCs (D) and lineage-positive cells (E). (F and G) Percentage of GFP^+^ cells in PBMCs. Shown is the percentage of GFP^+^ cells in total PBMC (F) and lineage-positive cells (G). *p < 0.05.

One potential reason for the low transgene marking rates in the periphery could be that most GFP^+^ HSCs in bone marrow are quiescent. The chemotherapy drug 5-fluorouracil (5-FU) is used to enrich bone marrow cells for HSCs by triggering HSCs proliferation.^16^ Based on the kinetics of loss of GFP marking in PBMCs (see Figure 1E), we decided to start 5-FU treatment at week 20 after *in vivo* transduction, when initially transduced PBMCs had nearly disappeared from the periphery. 5-FU injections were continued weekly for 10 weeks. The cyto-depleting effect of 5-FU on lin^+^ cells in bone marrow, spleen, and PBMCs was observed based on the decline in the percentage of CD19^+^ cells in mononuclear cells (MNCs) (Figure S7). We then compared GFP marking before the start of 5-FU treatment (week 20) with that at week 30 (Figure 4). In bone marrow, upon 5-FU treatment, the percentage of GFP^+^ cells significantly increased in total MNCs, CD3^+^, and CD19^+^ lineage cells, while it remained unchanged in Gr-1^+^ and LSK cells (Figure 4A–4C). 5-FU treatment did not significantly change GFP marking in the spleen, in total MNCs, or in lin^+^ cells and remained in the range of 10%–20% (Figures 4D and 4E). As a result of 5-FU treatment, GFP expression became detectable in PBMCs and was in the range of ∼3% (Figure 4F). Marking increased in all three peripheral blood cell lineages analyzed (Figure 4G). These results support our hypothesis that forcing bone marrow GFP^+^ HSCs to proliferate stimulates their differentiation and exit from the bone marrow and increases transgene marking in peripheral blood cells.

**Figure 5:**
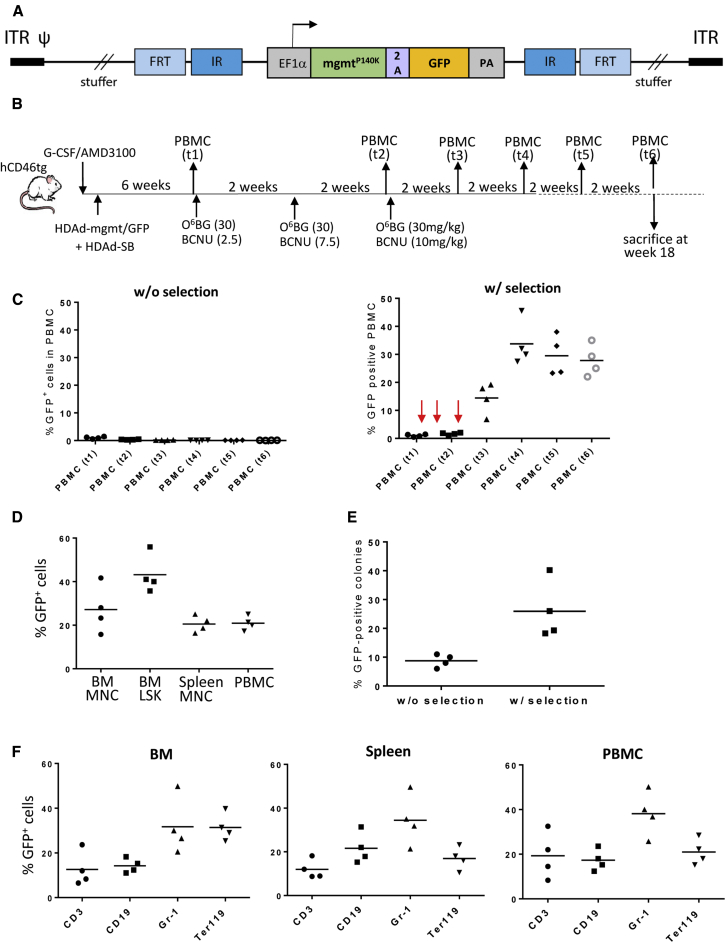
*In Vivo* Selection of *In Vivo*-Transduced HSCs (A) Vector design. The mgmt^P140K^ gene is linked to the GFP gene through a self-cleaving picornavirus 2A peptide. Both genes are under the control of the EF1α promoter. PA, poly-adenylation signal. The transgene cassette is flanked by inverted repeats (IR), which are recognized by the SB100x transposase, and frt sites, which are recognized by the Flp recombinase. (B) Treatment regimen. hCD46tg mice were mobilized and i.v. injected with HDAd-mgmt/GFP + HDAd-SB (2 times each, 4 × 10^10^ vp of a 1:1 mixture of both viruses). 4 weeks later, PBMCs were harvested before O^6^-BG/BCNU treatment was started (time point “PBMC [t1]”). This cycle was repeated every 2 weeks for a total of 3 times. With each cycle, the BCNU concentration was increased from 2.5 mg/kg to 7.5 mg/kg and 10 mg/kg. GFP marking was followed until week 18, when animals were sacrificed for GFP analysis in other tissues. (C) Percentage of GFP^+^ PBMCs in mice without O^6^-BG/BCNU treatment (left panel) and with drug (right panel). O^6^-BG/BCNU treatment is indicated by vertical red arrows. The PBMC collection time points (“PBMC [t1] to PBMC [t6]” are shown in B). (D–F) Week 18 analyses. Percentage of GFP^+^ MNCs and LSK cells in bone marrow (BM), splenic MNCs, and PBMCs (D). GFP^+^ colonies. Total bone marrow lin^−^ cells were plated, and GFP expression in colonies was analyzed 12 days later. Each symbol is the average GFP^+^ colony number for individual mice. The total number of colonies that developed was comparable for mice with and without *in vivo* selection (E). GFP^+^ cells in CD3, CD19, Gr-1, and Ter119 lineage-positive cells in bone marrow, spleen, and PBMCs (F).

Treatment with 5-FU over extended time periods to increase gene marking in PBMCs is not possible in patients because of potential immunosuppressive side effects. We therefore evaluated the mgmt^P140K^ *in vivo* selection approach, which requires only short-term exposure to O^6^-BG/BCNU at doses neither immunosuppressive nor myeloablative.^17^ We expected that this would give GFP^+^ HSCs a proliferation stimulus and support the selective survival and expansion of progeny cells. For *in vivo* selection, we modified our HDAd-GFP transposon vector by linking the mgmt^P140K^ gene to the GFP gene via a self-cleaving picornavirus 2A peptide (Figure 5A). We first showed *in vitro* that the corresponding HDAd5/35++ vector (HDAd-mgmt/GFP) conferred resistance to O^6^-BG/BCNU (Figure S8). We then tested a combined *in vivo* HSC transduction/*in vivo* HSC selection approach in hCD46tg mice (Figure 5B). Animals were mobilized with G-CSF/AMD3100 and intravenously injected with HDAd-mgmt/GFP plus HDAd-SB. GFP expression in PBMCs at 6 weeks after transduction (before the first O^6^-BG/BCNU cycle) was 1.2 (±0.4)% (Figure 5C, time point “t1”). In two animals sacrificed at this time point, 5.3 and 6.1% of bone marrow LSK cells expressed GFP. This is in the range published previously.^1^ In the remaining mice,6 weeks after *in vivo* transduction, three drug treatment cycles (with 30 mg/kg O^6^-BG and increasing BCNU doses) were given at 2-week intervals (Figure 5B). The control group was transduced *in vivo* but did not receive O^6^-BG/BCNU. In this group, the percentage of GFP^+^ PBMCs was less than 1% (Figure 5C, left panel). In the group that received *in vivo* selection drugs, the percentage of GFP^+^ PBMCs started to increase after the second treatment and reached on average of 35% GFP^+^ PBMCs 4 weeks after the last drug injection (Figure 5C, right panel). At week 18 after *in vivo* transduction (week 8 after the last drug treatment), on average 25% of PBMCs expressed GFP. At this time point, the average percentage of GFP^+^ MNCs in mice was 28% in bone marrow and 20% in spleen and PBMCs (Figure 5D). About 45% of bone marrow LSK cells were GFP^+^, indicating that transduced LSK cells had also expanded (Figure 5D). In colony assays with plated bone marrow lin^−^ cells, 30% of colonies were GFP^+^ (Figure 5E). Analysis of GFP marking in CD3, CD19, Gr-1, and Ter119 lin^+^ cells in bone marrow, spleen and blood showed comparable levels in the range of 10%–40% (Figure 5F). This suggests transduction of primitive HSCs with our integrating HDAd-mgmt/GFP vector and multi-lineage expansion. In mice that did not receive O^6^-BG/BCNU the percentage of stably transduced lin^−^ cells in bone marrow ranged from 2% to 5%, a finding that is in agreement with our previous study (Figure S9; data not shown).

To assess side effects of O^6^-BG/BCNU on hematopoiesis and differentiated blood cells, we analyzed the cellular composition of bone marrow, spleen, and blood at week 18 after *in vivo* transduction (Figure S10A). Compared to untreated control mice (Figure S10B), no remarkable differences were found. We also analyzed the acute effect of O^6^-BG/BCNU. In the peripheral blood, a significant decrease in total white blood cell, neutrophil, lymphocyte, and platelet counts occurred between days 7 and 10 after drug injection at BCNU doses greater than 5 mg/kg (Figure S11). Blood cell counts were restored to physiological levels by day 14 post-drug injection. In bone marrow, at day 3 after O^6^-BG/BCNU (10 mg/kg) treatment, the percentage of Gr-1^+^ cells was 50% of levels seen in untreated mice (Figure S12). Furthermore, we observed an increase in CD3^+^, CD19^+^, and Ter119^+^ cells at day 3. Cell composition in bone marrow at week 4 after O^6^-BG/BCNU treatment was comparable to untreated animals. This indicates the absence of a major cyto-depleting effect caused by 10 mg/kg BCNU in bone marrow.

**Figure 6:**
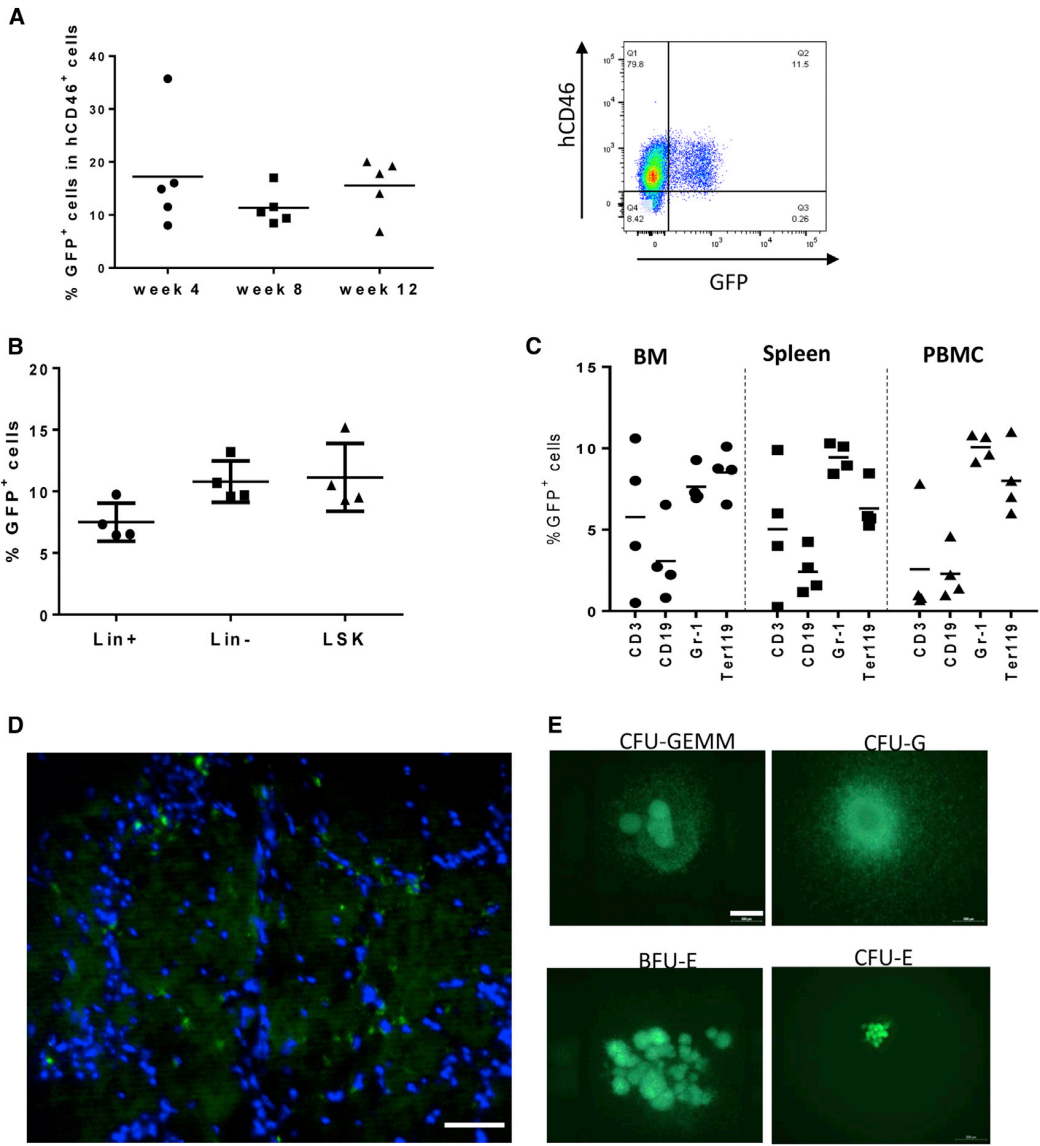
Analysis of GFP Marking in Secondary Recipients (A–E) Bone marrow cells from mice shown in Figure 5 were harvested at week 18 after *in vivo* transduction of hCD46tg mice, depleted for lineage-positive cells, and transplanted into lethally irradiated C57BL/6 mice. (A) Flow cytometry of PBMCs at weeks 4, 8, and 12 after transplantation in four recipient mice. The right panel shows a typical analysis. The vertical axis shows staining for hCD46, and the horizontal axis shows GFP staining. (B) GFP flow analysis of bone marrow LSK, lineage-positive, and lineage-negative cells in recipients 8 weeks after transplantation. (C) Analysis of GFP expression in bone marrow, spleen, and peripheral blood T-, B-, myeloid, and erythroid lineage-positive cells. (D) GFP immunofluorescence of brain tissue sections from recipients. GFP^+^ resemble microglia. Nuclei are stained with DAPI (blue). The scale bar represents 50 μm. (E) GFP expression in progenitor colonies. Examples for GFP^+^ burst-forming units-erythroid (BFU-E), colony-forming units of erythroid progenitors (CFU-E), granulocyte progenitors (CFU-G), granulocyte/macrophage progenitors (CFU-GM), and multi-potential progenitor cells CFU-GEMM (granulocyte, erythrocyte, monocyte, and megakaryocyte) are shown. Scale bar represents 500 μm.

To further confirm that our combined HSC *in vivo* transduction/selection approach resulted in genetic modification of primitive HSCs, we performed a transplantation/repopulation study. Lineage^−^ bone marrow cells from hCD46tg mice collected at week 18 after *in vivo* transduction/selection were used for transplantation into lethally irradiated C57BL/6 mice. The percentage of GFP^+^ cells in the lin^−^ cell transplant (pooled from 3 mice) was 40%. Analysis of hCD46 expression on PBMCs at weeks 4, 8, and 12 after transplantation showed engraftment rates of >90% in all recipients. The percentage of GFP^+^/hCD46^+^ cells was stable and on average 15% (Figure 6A). In bone marrow, GFP marking at week 12 was also ∼12% in LSK cells, lin^−^ and (total) lin^+^ cells (Figure 6B). A comparable marking rate was seen in Gr-1^+^ and Ter119^+^ cells in bone marrow, spleen, and PBMCs (Figure 6C); however, variations between animals in GFP^+^ CD3^+^ and CD19^+^ cells were larger. This could imply that our vector system more efficiently transduced common myeloid progenitors than common lymphoid progenitors. GFP^+^ cells were also observed in the brain (Figure 6D), lung, liver, and kidney (not shown) most likely originating from GFP^+^ HSCs. Bone marrow lin^−^ cells from recipients gave rise to ∼10% GFP^+^ colonies. GFP^+^ colonies included CFU-GEMM, CFU-G, BFU-E, and CFU-E expressing GFP (Figure 6E).

**Figure 7:**
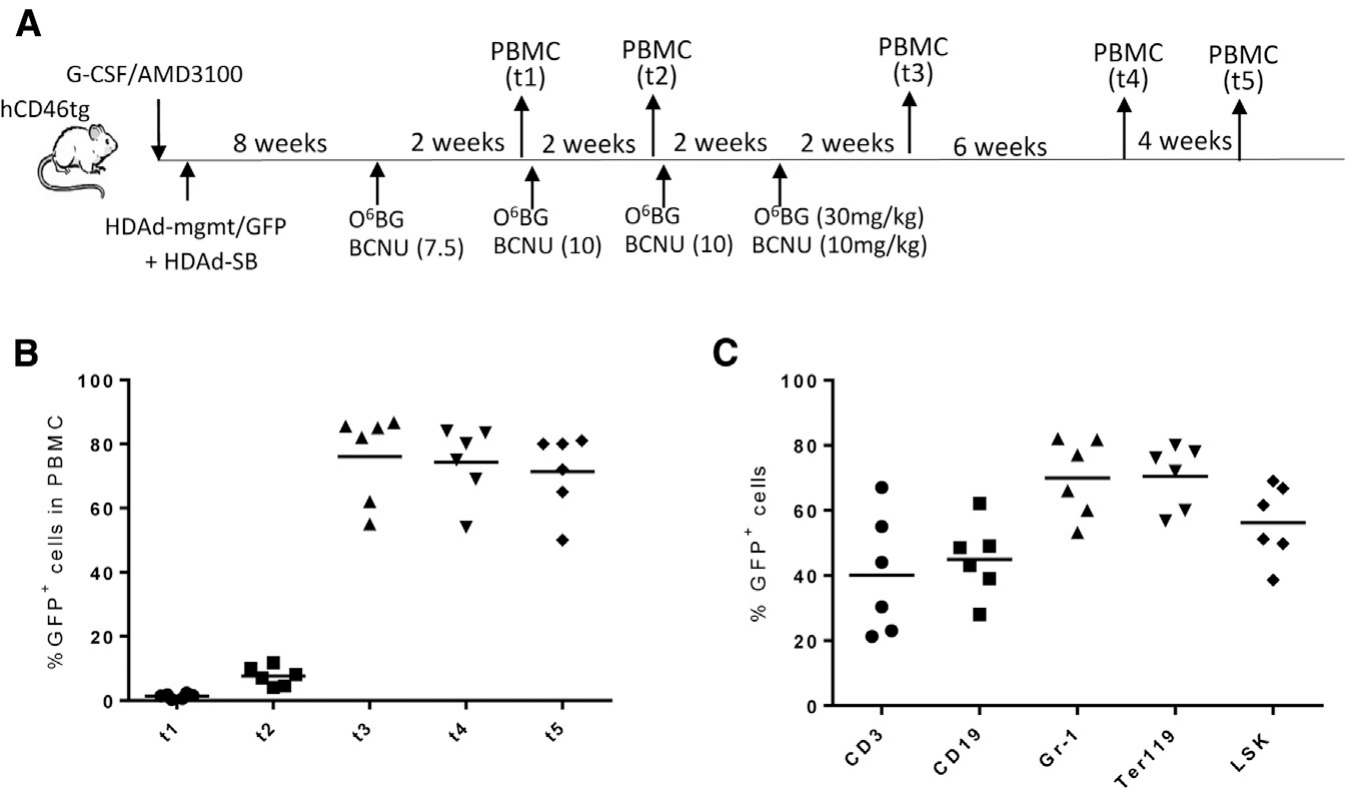
*In Vivo* Transduction in Combination with Additional Rounds of Drug Selection and Subsequent Immunosuppression (A) Treatment regimen. hCD46tg mice were mobilized and i.v. injected with HDAd-mgmt/GFP + HDAd-SB (2 times each, 2 × 10^10^ vp of a 1:1 mixture of both viruses). 8 weeks later, *in vivo* selection was started with a dose of 30 mg/kg O^6^-BG + 7.5 mg/kg BCNU, followed by three rounds of O^6^-BG + 10 mg/kg BCNU cycles 2 weeks apart. Time points of PBMC collection are indicated. (B) Percentage of GFP^+^ PBMCs at the time points indicated in (A). (C) Analysis of GFP expression in bone marrow LSK and lineage-positive cells at time point “t5.”

To further increase transgene marking in PBMCs, we performed a study with two additional cycles of drug treatment (Figure 7A). Because we did not observe a cyto-depleting effect with BCNU doses below 5 mg/kg (see Figure S11), we started the *in vivo* selection process at a dose of 7.5 mg/kg followed by three doses of O^6^-BG 10 mg/kg BCNU 2 weeks apart. 2 weeks after the last drug injection, GFP marking in PBMCs reached >80% and remained stable (Figure 7B). At the last time point analyzed (t5), 12 weeks after the last drug injection, GFP expression was seen on average in 40% CD3^+^, 40% CD19^+^, 70% Gr-1^+^, and 70% Ter119^+^ of bone marrow lineage cells and in ∼50% of LSK cells. Overall, our second *in vivo* HSC transduction/selection study illustrates the power of the approach for reaching high transgene marking levels in peripheral blood cells. Notably, compared to previous studies with the mgmt *in vivo* selection system, our approach does not require the isolation of HSCs and subsequent transplantation.

## Discussion

Current protocols for HSC gene therapy, involving the transplantation of *ex vivo* lentivirus vector-transduced HSCs into myelo-ablated recipients, are complex, expensive, and therefore difficult to perform in countries with limited resources. We developed a simplified approach for *in vivo* HSC gene therapy that does not require myeloablation and HSC transplantation. In our previous study, we have shown that this approach was safe and resulted in efficient, long-term transgene expression in primitive HSCs. While we found high GFP marking rates in bone marrow HSCs, the frequency of GFP-expressing PBMCs was below 1% and did not increase over time. It is possible that even this low transgene marking rate is sufficient in diseases in which gene corrected HSC/progeny have a proliferative advantage over non-transduced cells such as X-linked SCID,^18^ ADA-SCID,^19^ and Fanconi anemia.^20^ However, low transgene marking in differentiated peripheral blood cells represents a major limitation of our approach for gene therapy of common genetic diseases, such as hemoglobinopathies, which require the correction of the majority of target cells.

We developed two (non-exclusive) hypotheses for the low transgene marking in peripheral blood cells: (1) the number of GFP^+^ HSCs that are transduced in the periphery and return to the bone marrow is insufficient, and (2) the majority of GFP^+^ HSCs in bone marrow are quiescent not contributing to downstream differentiation.

Our first hypothesis that increasing the percentage of transduced HSCs in bone marrow by using more selective mobilization drugs would be reflected in increased gene marking in peripheral blood cells did not prove to be correct. We believe however that our data are of interest for a better understanding of our *in vivo* transduction approach and of the fate of HSCs after mobilization with different drugs: (1) G-SCF alone and in combination triggered pronounced leukocytosis; (2) our *in vivo* HSC marking approach indicates that AMD3100 and BIO5192 results in more efficient *in vivo* transduction of HSCs/progenitors that return to the bone marrow; (3) at week 8 after *in vivo* transduction, HDAd genomes can still exist in episomal form in HSCs; and (4) SB100x-mediated integration requires an effect triggered by G-CSF. The latter needs to be studied in the future.

The second hypothesis that GFP^+^ HSCs in bone marrow are dormant and need to be pushed into cycling is supported by our studies with 5-FU and the mgmt *in vivo* selection system. Five rounds of treatment resulted in >80% of stable PBMC marking rates in all lineages, including erythroid cells. Furthermore, our data indicate the transduction of primitive, long-term persisting HSC that survive *in vivo* selection and drive the repopulation of the hematopoietic system after transplantation into lethally irradiated recipients. That the pool of GFP^+^ HSCs is maintained suggests that the *in vivo* selection approach stimulates assymetric cell division in GFP^+^ HSCs. The finding that the percentage of GFP^+^ LSK cells increases upon *in vivo* selection suggests that the increased GFP marking in PBMCs is not only due to cell cycle changes in HSCs but also due to a selection advantage.

Others and our group previously reported that GFP-specific T cells can develop in HSC gene therapy setting.^1^, ^21^ This could be a third element that contributes to low GFP marking in the periphery without *in vivo* selection. Notably, immune-responses against a therapeutic transgene product are absent when the transgene is from the same species. MGMT is a human protein and it is expected that there is also tolerance to the MGMT^P140K^ mutant in humans.^17^

Our studies were performed in an adequate mouse model (hCD46tg mice) that expressed the HDAd5/35++ receptor CD46 in a pattern and at a level similar to humans.^22^ We previously showed that hCD46 in these mice is expressed on all bone marrow HSCs.^1^ Notably, to bridge our studies in hCD46tg mice and potential studies in humans, we did not plan to use humanized mice generated by sublethal irradiation of immunodeficient NSG mice and transplantation of human CD34^+^ cells. These mice have abnormal bone marrow structure, hematopoiesis, and are difficult to mobilize.^23^ To translate our approach into the clinic, we will focus on efficacy studies on mouse models for human diseases (e.g., β-thalassemia or Fanconi anemia) and safety studies in non-human primates. Importantly, we have used the mgmt-based *in vivo* HSC selection approach using temozolomide (in the context of lentivirus vectors) in patients with glioblastoma.^15^, ^24^ Furthermore, we have an ongoing clinical study using it to increase HIV resistant cells *in vivo*. Therefore, this approach is clearly feasible and thus highly relevant for hemoglobinopathies.

In none of the long-term follow-up studies involving *ex vivo* lentivirus vector-transduced HSCs and mgtm-based *in vivo* selection in dogs or non-human primates, clonal dominance or leukemic events have been found.^11^, ^14^ The SB100x transposase system confers random transgene integration into the genome of HSCs.^1^ From a genotoxicity standpoint, this is, theoretically better than currently used lentivirus or rAAV vectors, which have preference for integrating into active genes. To address concerns of potential activation of proto-oncogenes with our *in vivo* HSC transduction/selection approach, we are planning longitudinal integration site studies in non-human primates.

In summary, our *in vivo* HSC transduction/selection is well tolerated and capable of achieving high transgene marking levels in peripheral blood cells. Importantly, our approach eliminates the need for HSC isolation, *in vitro* culture and transplantation and should therefore make HSC gene therapy more accessible to patients.

## Materials and Methods

### Reagents

G-CSF (Neupogen) fom Amgen (Thousand Oaks, CA, USA), AMD3100 from Sigma-Aldrich (St. Louis, MO, USA), BIO5192 from Tocris (Bristol, UK), and heparin from Fresenius Kabi (Lake Zurich, IL, USA) were used. BOP was provided by CSIRO (Clayton, VIC, Australia). O^6^-BG and BCNU were from Sigma-Aldrich (St, Louis, MO, USA).

### HDAd Vectors

Stable transduction studies were performed with helper-dependent HDAd5/35++ vectors using a hyperactive Sleeping Beauty transposase (SB100x) system for integration of the GFP transgene cassette.^3^, ^25^ This vector systems consists of two vectors. The transposon vector (HDAd-GFP) carries a GFP expression cassette that is flanked by inverted transposon repeats (IR) and *frt* sites. The second vector (HDAd-SB) provides both Flpe recombinase and SB100x transposase in *trans*.^1^ The HDAd-GFP/mgmt vector was constructed as follows: the EF1a-mgmt(P140K)-2a-GFP-pA was synthesized by Genscript (Nanjing, China). The cassette was released by ICeu-I, blunted, and inserted into the Pme I site of pHM5-FIP between the two Sleeping Beauty IRs. The complete transposon cassette was then released by PI-SceI and I-CeuI digest and introduced into pHCA via recombineering.^26^ For the production of HDAd-SB and HDAd-mgmt/GFP virus, corresponding plasmids were linearized with *PmeI* and rescued in 116 cells^27^ with AdNG163-5/35++, an Ad5/35++ helper vector containing chimeric fibers composed of the Ad5 fiber tail, the Ad35 fiber shaft, and the affinity-enhanced Ad35++ fiber knob.^1^ Helper virus contamination levels were found to be <0.05%. All preparations were free of bacterial endotoxin. Titers were 3 to 9 × 10^12^ viral particles (vp)/mL.

### Mobilization and *In Vivo* Transduction

All experiments involving animals were conducted in accordance with the institutional guidelines set forth by the University of Washington. The University of Washington is an Association for the Assessment and Accreditation of Laboratory Animal Care International (AALAC)-accredited research institution, and all live animal work conducted at this university is in accordance with the Office of Laboratory Animal Welfare (OLAW) Public Health Assurance (PHS) policy, USDA Animal Welfare Act and Regulations, the Guide for the Care and Use of Laboratory Animals, and the University of Washington's Institutional Animal Care and Use Committee (IACUC) policies. The studies were approved by the University of Washington IACUC (Protocol No. 3108-01.

HSCs were mobilized in mice by subcutaneous (s.c.) injection of human recombinant G-CSF (5 μg/mouse/day, 4 days) followed by an s.c. injection of AMD3100 (5 mg/kg) on day 5. In addition, animals received dexamethasone (10 mg/kg) i.p. 16 and 2 hr before virus injection. 30 and 60 min after AMD3100, animals were intravenously injected with a 1:1 mixture of HDAd-mgtm/GFP and HDAd-SB through the retro-orbital plexus with a dose of 4 × 10^10^ vp per injection (animals in Figures 1, 5, and 6). The animals in Figures 3 and 7 received 2 × 10^10^ vp per injection.

#### *In Vivo* Selection

hCD46tg mice were mobilized and intravenously (i.v.) injected with HDAd-mgtm/GFP + HDAd-SB. 4 weeks later, O^6^-BG/BCNU treatment was started. Mice were injected with O^6^-BG (15 mg/kg, intraperitoneal [IP]) 2 times, 30 min apart. 1 hr after the second injection of O^6^-BG, mice were injected with BCNU (2.5 mg/kg, IP). The BCNU dose was increased in the second and third cycles to 7.5 and 10 mg/kg, respectively.

### qPCR for Ad Genomes

Genomic DNA (gDNA) was isolated using the Blood and Tissue Kit (QIAGEN, Valencia, CA, USA) in accordance with the manufacturer’s instructions, and gDNA concentration was determined spectrophotometrically. gDNA samples were analyzed for vector genomes carrying a GFP cassette with the following primers: FWD: TCGTGACCACCCTGACCTAC, REV: GGTCTTGTAGTTGCCGTCGT. qPCR was performed using the Kapa SYBR Fast qPCR Kit Master Mix (Kapa Biosystems, Boston, MA, USA). Each reaction was run in triplicates. Serial dilutions of purified HDAd-GFP viral DNA were used as a standard curve.

### Flow Cytometry

Cells were resuspended at 1 × 10^6^ cells/100 μL in PBS supplemented with 1% fetal calf serum (FCS) and incubated with Fc receptor (FcR) blocking reagent (Miltenyi Biotech, Auburn, CA, USA) for 10 min on ice. Next, the staining antibody solution was added in 100 μL per 10^6^ cells and incubated on ice for 30 min in the dark. After incubation cells were washed once in fluorescence-activated cell sorting (FACS) buffer. For secondary staining, the staining step was repeated with a secondary staining solution. After the wash, cells were resuspended in FACS buffer and analyzed using a LSRII flow cytometer (BD Biosciences). Debris was excluded using a forward scatter-area and sideward scatter-area gate. Single cells were then gated using a forward scatter-height and forward scatter-width gate. Flow cytometry data were then analyzed using FlowJo (v.10.0.8).

The following flow cytometry antibodies and reagents were used: lineage cell detection cocktail-biotin (mouse; Miltenyi Biotec); streptavidin allophycocyanin (APC; eBioscience, San Diego, CA, USA); anti-mouse LY-6A/E (Sca-1), PE-Cyanine7, clone D7 (eBioscience); anti-mouse CD117 (c-Kit)-PE, Clone 2B8 (eBioscience); anti-Mouse CD3^−^ APC, Clone 17A2 (eBioscience); anti-mouse CD19-PE-Cyanine7, Clone eBio1D3 (eBioscience); anti-mouse Ly-66 (Gr-1)-PE, Clone RB6-8C5 (eBioscience); and anti-mouse TER-119-APC, Clone TER-119 (Biolegend, San Diego, CA, USA).

### Magnetic Cell Sorting

For the depletion of lineage-committed cells, the Mouse Lineage Cell Depletion Kit (Miltenyi Biotec, San Diego, CA, USA) was used in accordance with the manufacturer’s instructions.

### CFU Assay

1,500 lineage^−^, GFP^+^ cells were plated in triplicates in ColonyGEL 1202 mouse complete medium (ReachBio, Seattle, WA, USA) and incubated for 12 days at 37°C in 5% CO_2_ and maximum humidity. Colonies were enumerated using a Leica MS 5 dissection microscope (Leica Microsystems). For scoring of GFP^+^ colonies, an Olympus IMT-2 UV microscope was used with a DFC 300 FX camera (Leica Microsystems).

### Tissue GFP Immunofluorescence Analysis

Before tissue harvest, blood was flushed from the circulation with PBS using the heart as a pump. Tissues were fixed in formalin overnight (o/n) and subsequently incubated for 2 hr in PBS and 5%, 10%, and 20% sucrose. Tissues were then embedded in optimal cutting temperature compound (OCT) and sectioned (6 mm). Sections were incubated with Vectashield containing DAPI (Vector Laboratories, Burlingame, CA, USA). Immunofluorescence microphotographs were taken at room temperature on a Leica DMLB microscope (Leica, Wetzlar, Germany), with a Leica DFC300FX digital camera and Leica Application Suite (v.2.4.1) R1 (Leica Microsystems, Heerbrugg, Switzerland). No specific feature within images shown in was enhanced, obscured, moved, removed, or introduced.

### Secondary Bone Marrow Transplantation

Recipients were 6- to 8-week-old female C57BL/6 mice. On the day of transplantation, recipient mice were irradiated with 1000 Rad. Bone marrow cells from *in vivo*-transduced hCD46tg mice were isolated aseptically, and lineage-depleted cells were isolated using magnetic cell sorting (MACS). 4 hr after irradiation, cells were injected intravenously through the tail vein at 1 × 10^6^ cells per mouse.

### Blood Cell Counts

Blood samples were collected into EDTA-coated tubes, and analysis was performed on a HemaVet 950FS machine (Drew Scientific, Waterbury, CT, USA)

### Statistical Analyses

For comparisons of multiple groups, one-way and two-way analysis of variance (ANOVA) with Bonferroni post-testing for multiple comparisons was employed. Statistical analysis was performed using GraphPad Prism (v.6.01) (GraphPad Software, La Jolla, CA, USA).

## Author Contributions

A.L. provided the conceptual framework for the study. H.W., M.R., and A.L. designed the experiments. H.W., M.R., N.P., C.L., J.L., D.P, and P.N. performed the experiments. S.K.N. provided critical material. A.E., Z.I., K.G.H., H.-P.K., and T.P. provided comments. A.L. wrote the manuscript.

## Conflicts of Interest

The authors declare no competing financial interests.

## Supplemental Information

Document S1. Figures S1–S12

Document S2. Article plus Supplemental Information
