# Supplementary material for: A Combined In Vivo HSC Transduction/Selection Approach Results in Efficient and Stable Gene Expression in Peripheral Blood Cells in Mice
Source: Mol Ther Methods Clin Dev. 2017 Nov 10;8:52–64. doi: 10.1016/j.omtm.2017.11.004 (PMC5722719; doi:10.1016/j.omtm.2017.11.004)
Supplement: Document S1. Figures S1–S12 [file mmc1.pdf]

OMTM, Volume *8*

Supplemental Information

A Combined *In Vivo* HSC Transduction/Selection Approach Results in Efficient and Stable Gene Expression in Peripheral Blood Cells in Mice

Hongjie Wang, Maximilian Richter, Nikoletta Psatha, Chang Li, Jiho Kim, Jing Liu, Anja Ehrhardt, Susan K. Nilsson, Benjamin Cao, Donna Palmer, Philip Ng, Zsuzsanna Izsvák, Kevin G. Haworth, Hans-Peter Kiem, Thalia Papayannopoulou, and André Lieber

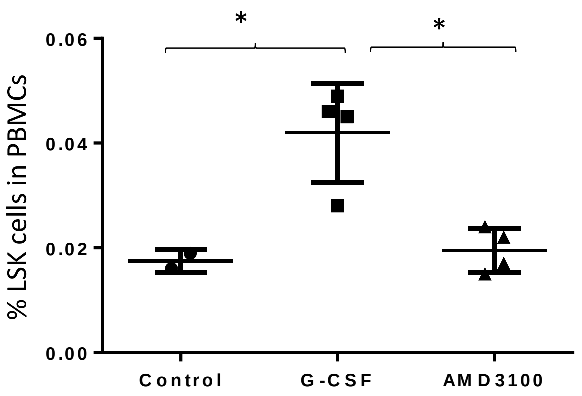

Fig.S1. Percentage of LSK cells in peripheral blood one hour after the last G-CSF injection or one hour after AMD3100 injection. Each symbol is one animal. * p<0.01

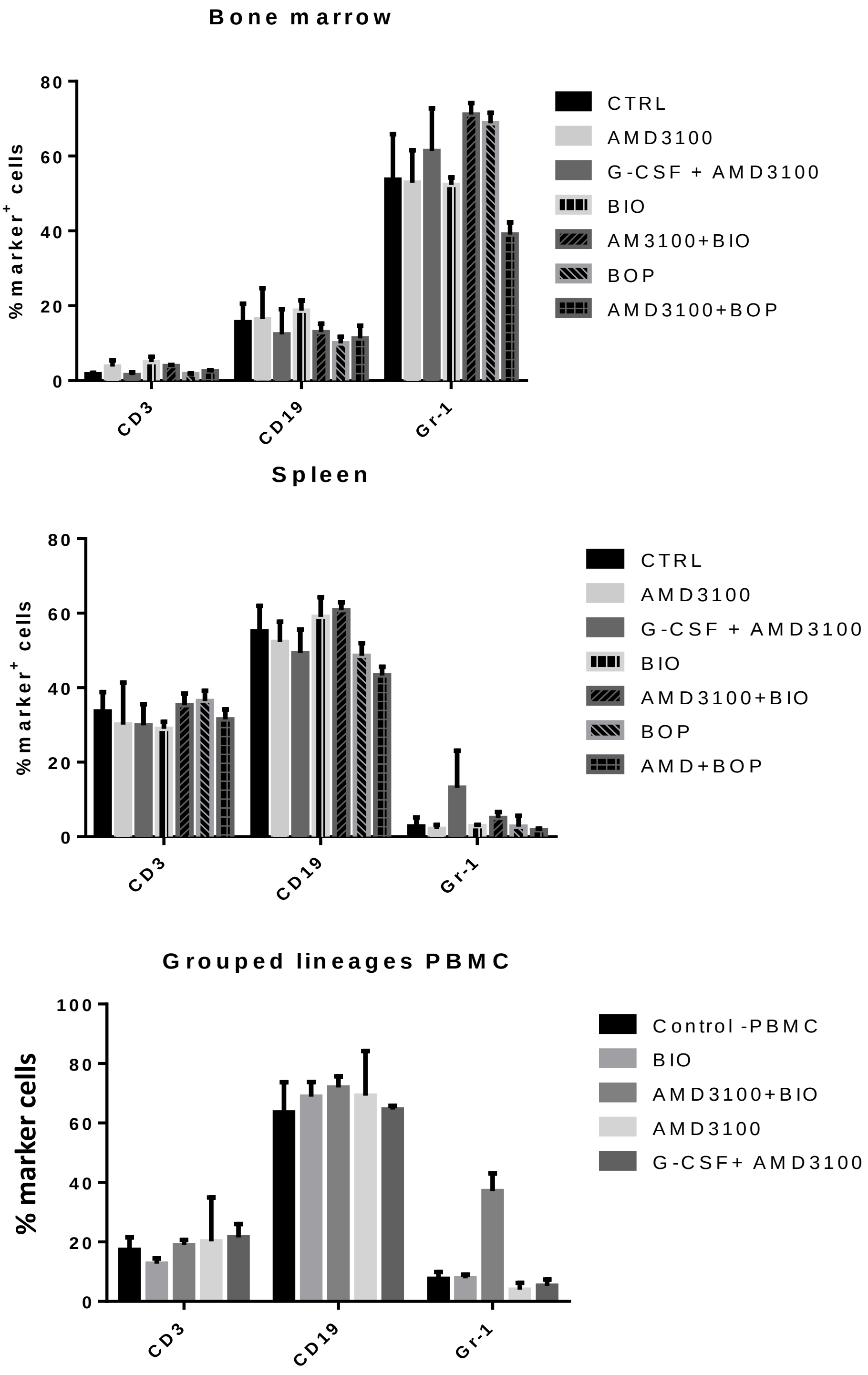

Fig.S2. Cellular composition of lineage-positive cells in bone marrow, spleen, and peripheral blood cells eight weeks after mobilization with the indicated drugs and *in vivo* transduction with HDAd-GFP + HDAd-SB. Mobilized groups were compared with a non-mobilized group ("control"). Shown is the percentage of $CD3^+$, $CD19^+$ and $Gr\text{-}1^+$ cells in all MNCs. The higher % of $Gr\text{-}1^+$ cells in G-CSG/AMD3100 mobilized animals is significant ($p<0.05$) and most likely due to granulocyte proliferation stimulated by G-CSF. BIO: BIO5192

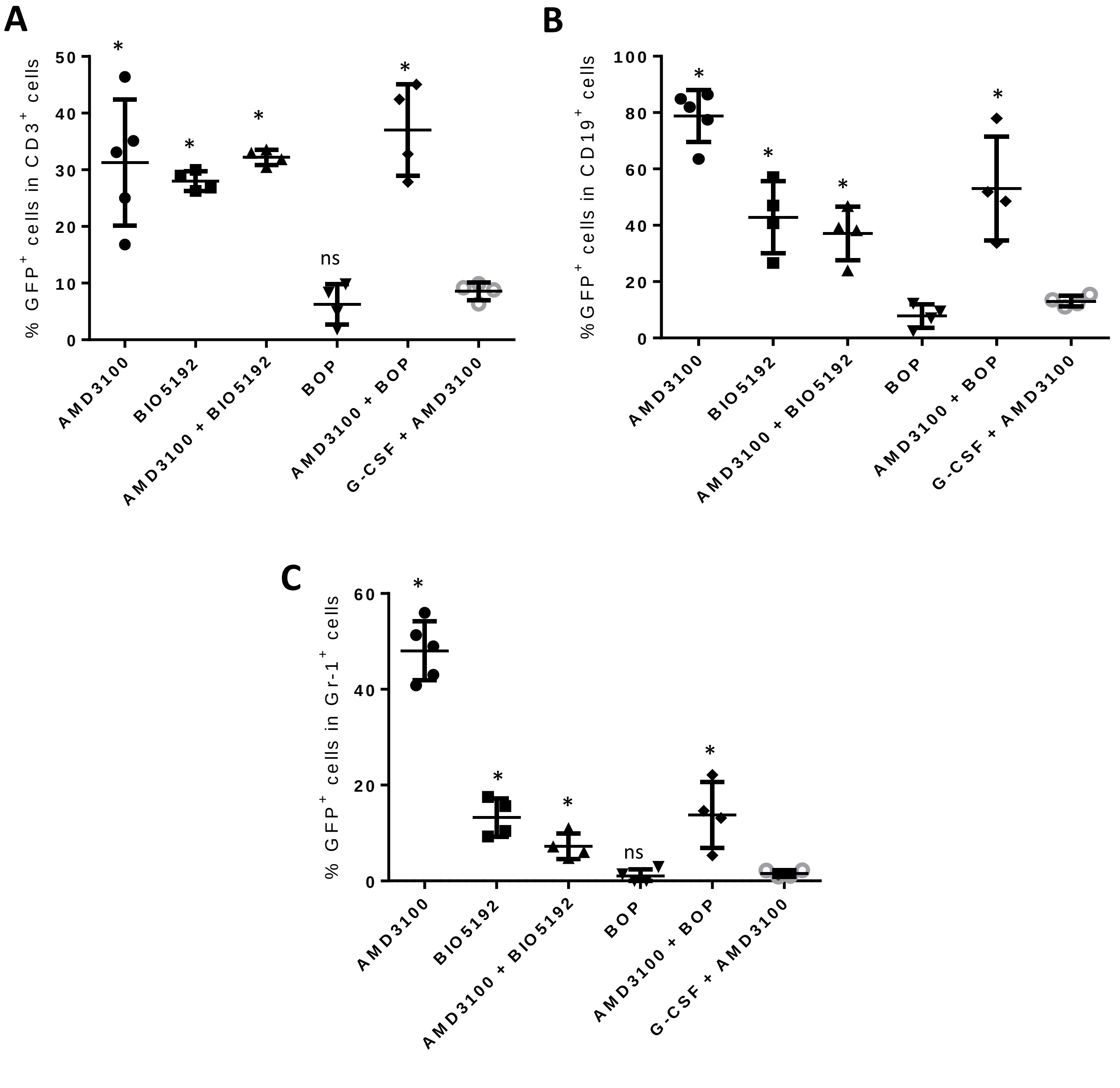

Fig.S3. GFP analysis in bone marrow lineage-positive cells 8 weeks after *in vivo* transduction with HDAd-GFP and HDAd-SB. hCD46tg mice were mobilized with the indicated agents and i.v. injected with HDAd-GFP + HDAd-SB. Mice were sacrificed at 8 after injection and bone marrow cells were harvested. **A**) Percentage of GFP$^+$ cells in CD3$^+$ lineage cells. **B)** Percentage of GFP$^+$ cells in CD19$^+$ lineage cells. **C)** Percentage of GFP$^+$ cells in Gr-1$^+$ lineage cells. * $p<0.05$ compared to G-CSF/AMD3100, ns – nonsignificant compared to G-CSF/AMD3100.

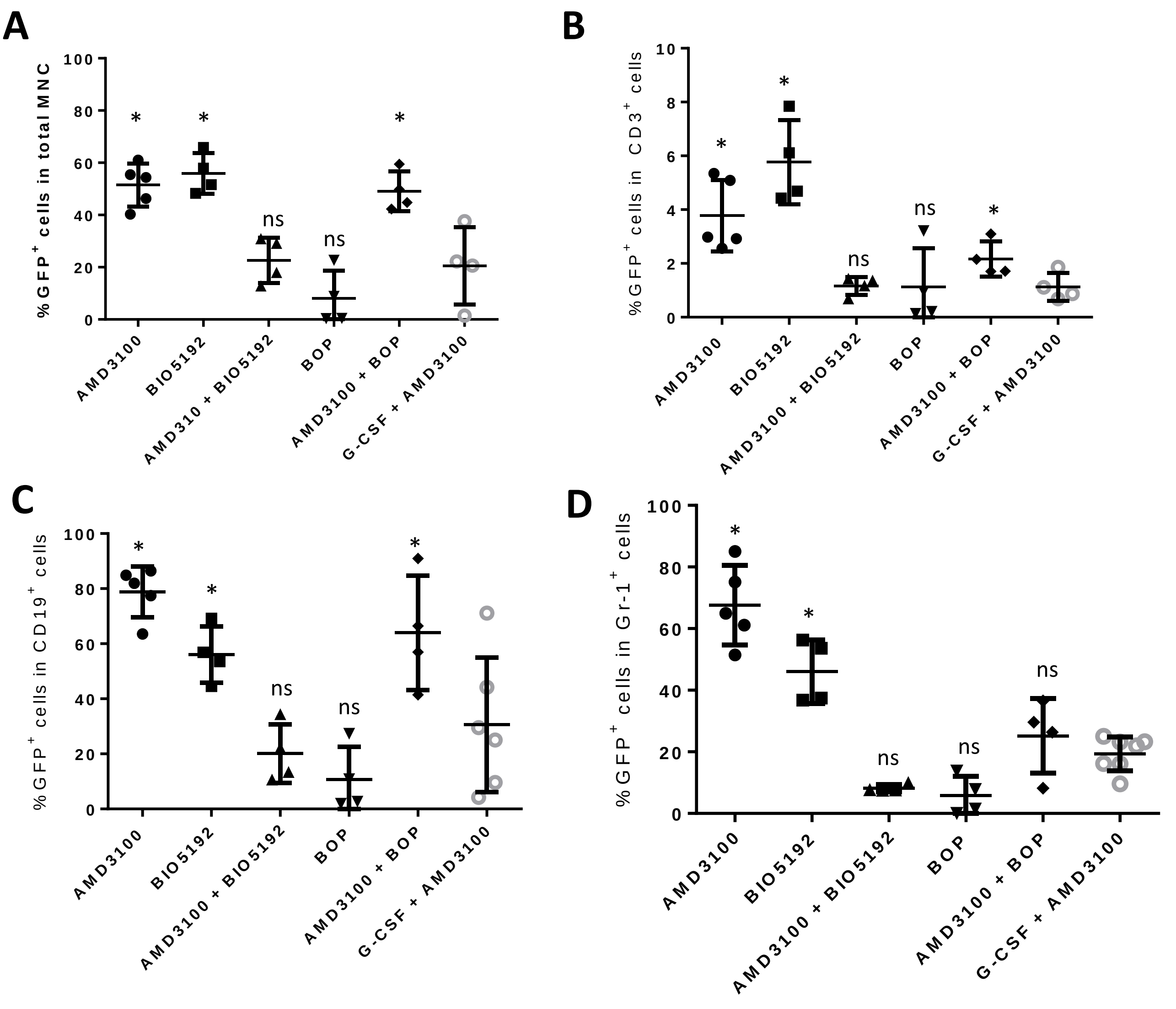

Fig.S4. GFP analysis in spleen 8 weeks after *in vivo* transduction with HDAd-GFP + HDAd-SB. The data are from the same animals analyzed for bone marrow cells in Fig.3. **A)** Percentage of GFP^+ cells in all splenic MNCs. **B)** Percentage of GFP^+ cells in all splenic $CD3^+$ cells. **C)** Percentage of GFP^+ cells in all splenic $CD19^+$ cells. **D)** Percentage of GFP^+ cells in all splenic $Gr\text{-}1^+$ cells. * $p<0.05$ compared to G-CSF/AMD3100, ns – nonsignificant compared to G-CSF/AMD3100.

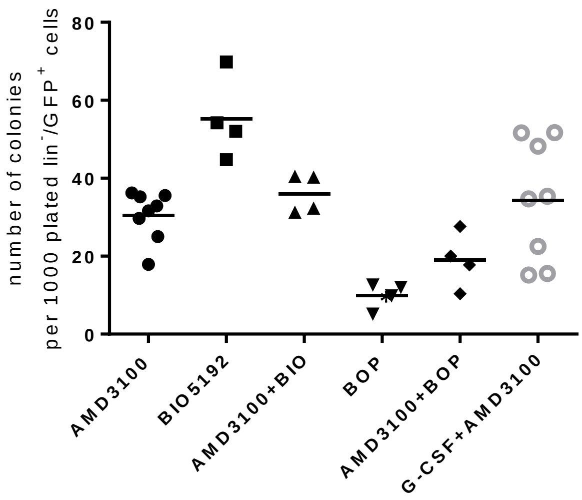

Fig.S5 Number of progenitor colonies that formed from 1000 plated lin$^-$/GFP$^+$ cells. Bone marrow MNCs harvested at 8 weeks after *in vivo* transduction were depleted for lineage-positive cells and sorted for GFP$^+$ cells.

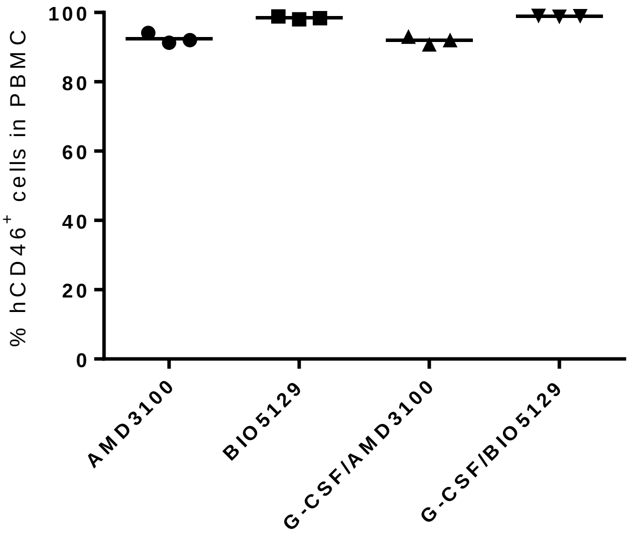

Fig.S6. Engraftment in secondary recipients. Lin^-/GFP^+ bone marrow cells harvested from *in vivo* transduced hCD46 transgenic were transplanted into C57Bl/6 mice. Shown is the percentage of $hCD46^+$ PBMCs in total PBMCs at week 8 after transplantation.

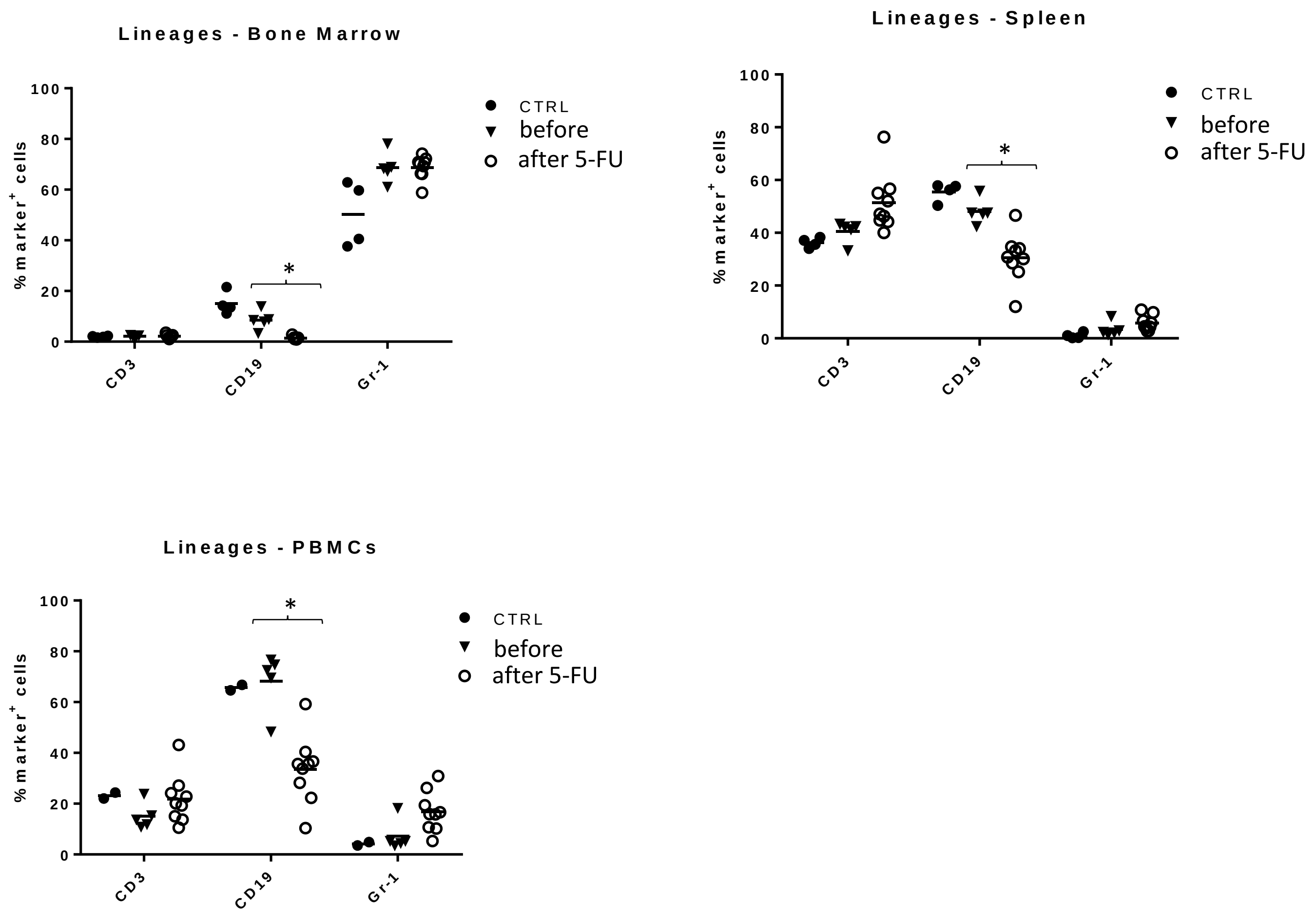

Fig.S7. Percentage of lineage-positive cells in all MNCs in the bone marrow, spleen, and PBMC before and after 5-FU treatment. The cyto-depleting effect of 5-FU is reflected in a decrease in $CD19^+$ cells in the bone marrow, spleen, and PBMC. * $p<0.05$

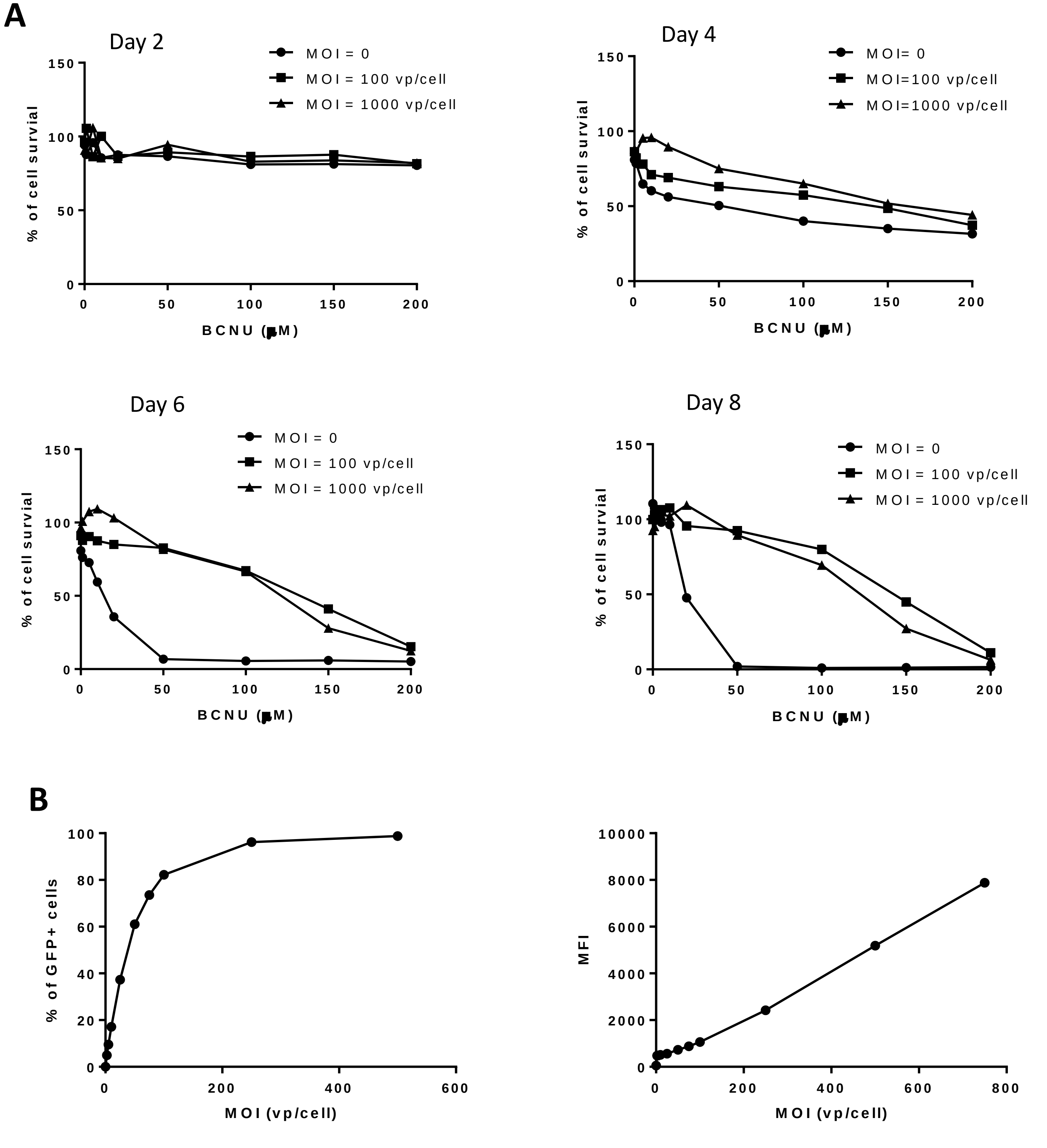

Fig.S8. Validation of HDAd-mgtm/GFP vector in vitro. A) mgmt^{P140K} confers resistance to 0^6BG/BCNU in vitro. Erythroleukemia K562 cells were mock-infected (MOI=0) or infected with HDAd-mgtm/GFP (MOI 100 and 1000 vp/cell). Twenty hours after infection, cells were incubated with 450µM O^6-BG for one hour and then exposed to BCNU at increasing concentrations for 2.5 hours. O^6-BG and BCNU were then washed off and cells were resuspended in fresh medium. Every 48 hours, cells were spun down and resuspended in fresh medium, 20% of cells were plated back. Viability in the remaining cells was measured by CellTiter 96® *AQueous* One Solution Cell Proliferation Assay (Promega), and normalized to control sample without $^{O6\text{-}BG}$/BCNU treatment in the corresponding group. **B)** HDAd-mgmt/GFP expresses the transgene GFP. K562 cells were infected at increasing MOIs and GFP was measured 24 hours later.

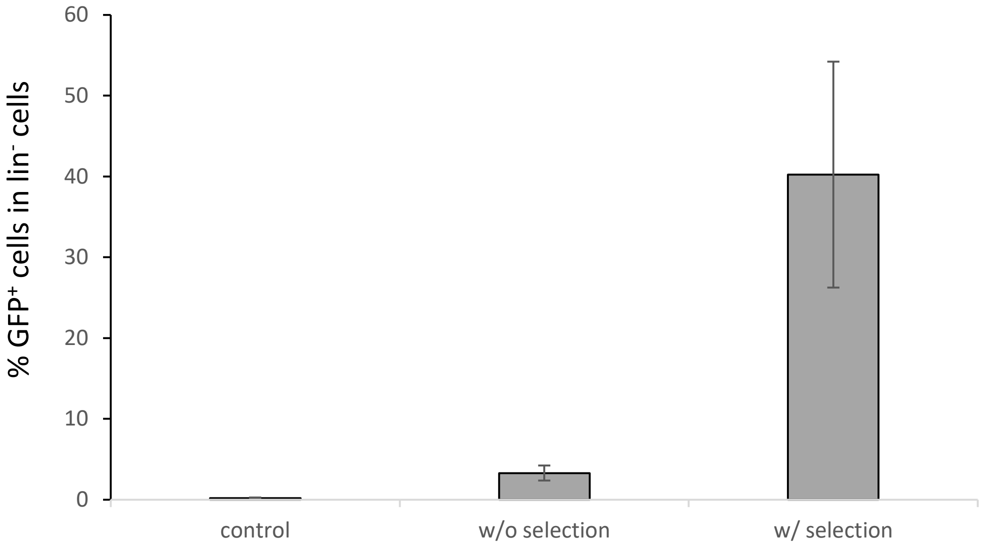

Fig.S9 Percentage of GFP+ lineage-negative cells in the bone marrow at week 18 after *in vivo* transduction. Compared were mice w/o HDAd injection (control), mice that received HDAd-mgmt/GFP plus HDAd-SB without drug selection and with drug selection. N=4.

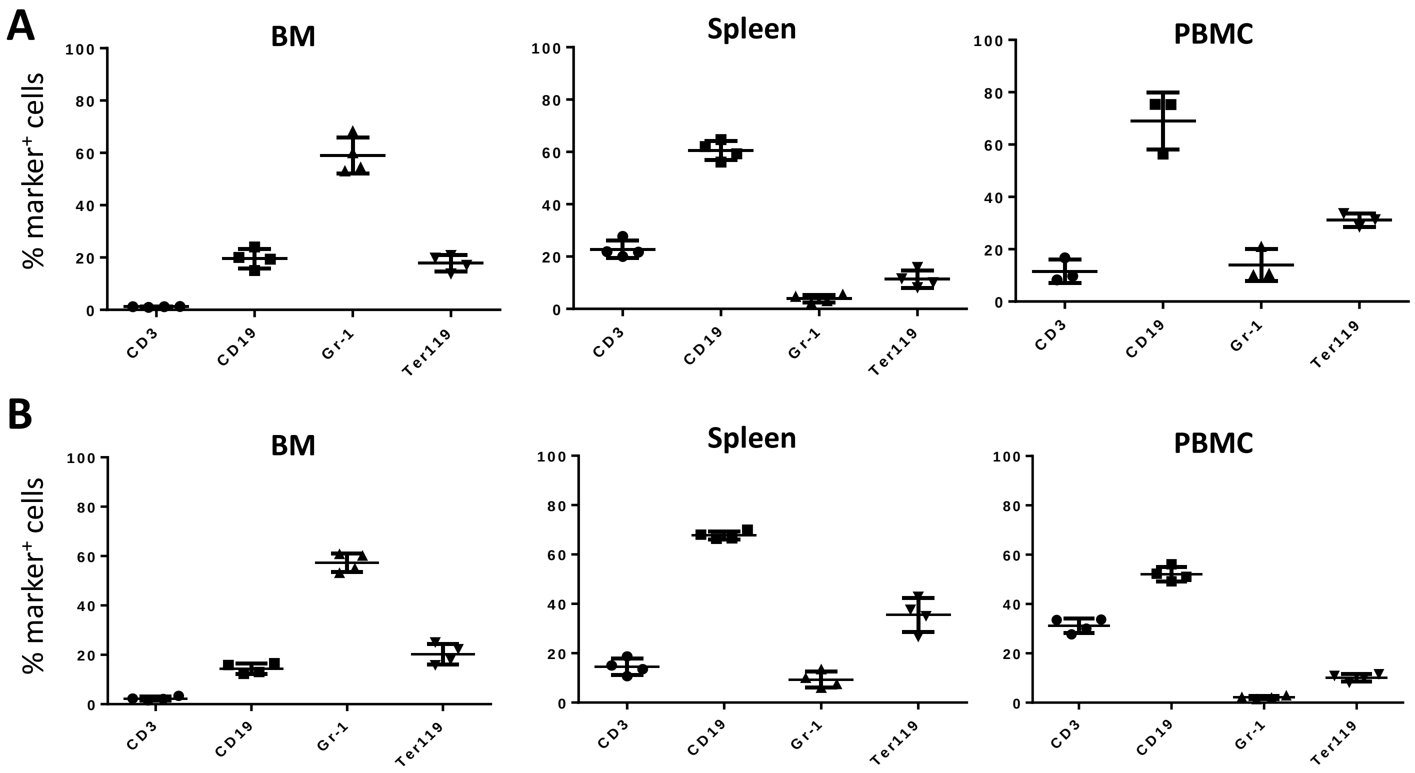

Fig.S10. Lineage cell composition in bone marrow, spleen and blood. A and B) Week 18 lineage-positive cell composition in bone marrow, spleen and blood. A) Mice treated with O^6-BG/BCNU. B) control mice (without *in vivo* transduction and selection).

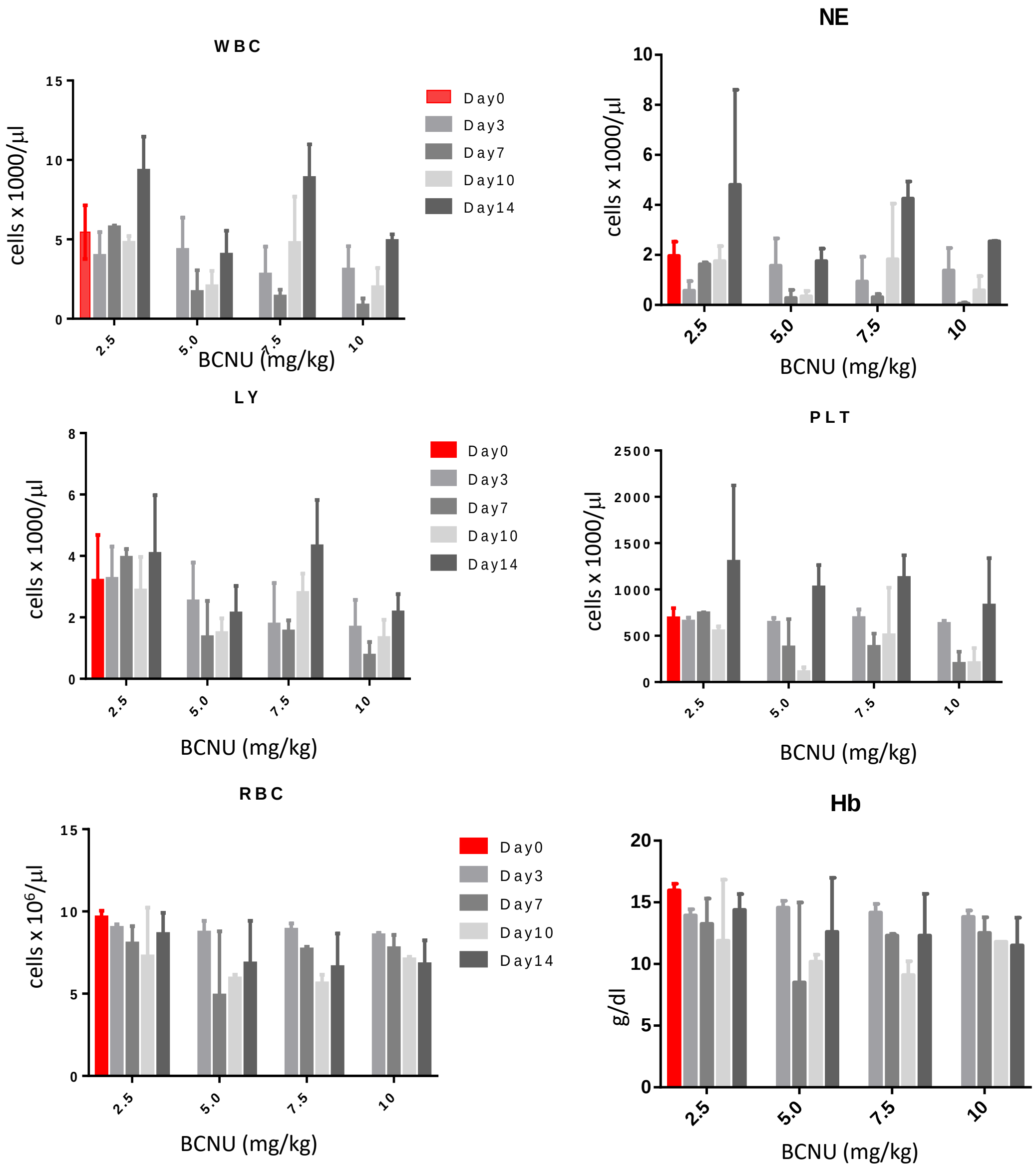

Fig.S11 Blood cell counts and hemoglobin levels in mice treated with O^6-BG/BCNU. hCD46 transgenic mice were treated once with O^6-BG (15mg/kg, IP) two times 30 minutes apart. One hour after second injection of O^6-BG, mice were injected with BCNU (2.5, 7.5, or 10mg/kg, IP). "Day 0" are pre-treatment levels. NE: neutrophils, LY: lymphocytes, PLT: platelets, RBC: red blood cells, Hb: hemoglobin

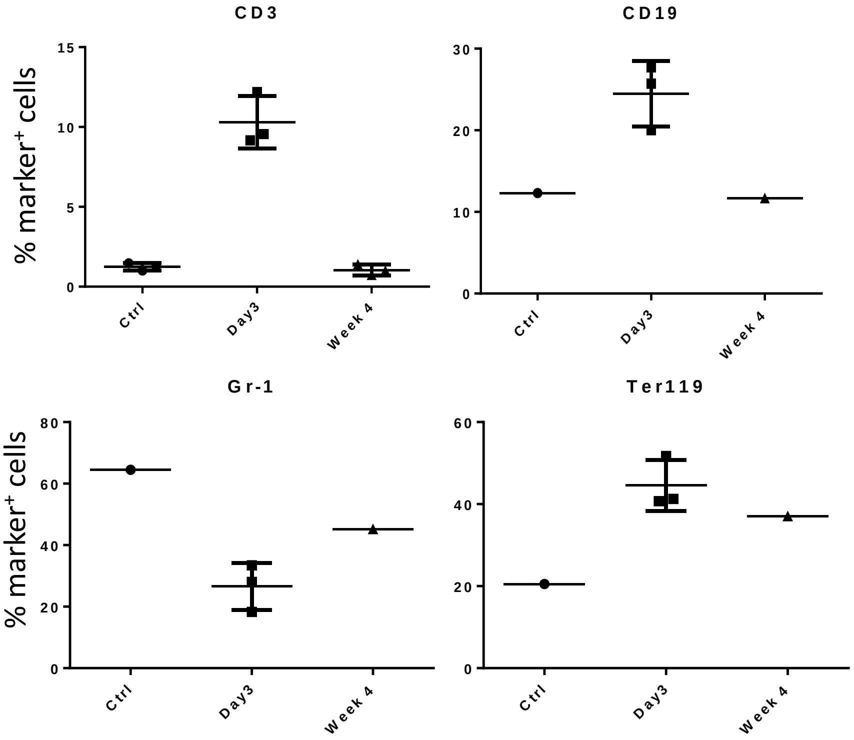

Fig.S12. Bone marrow lineage cell composition at day 3 and week 4 after 30mg/kg O^6-BG + 10mg/kg BCNU. Ctrl: animals that did not receive *in vivo* transduction/selection.
